# Red Blood Cell Function and Dysfunction: Redox Regulation, Nitric Oxide Metabolism, Anemia

**DOI:** 10.1089/ars.2016.6954

**Published:** 2017-05-01

**Authors:** Viktoria Kuhn, Lukas Diederich, T.C. Stevenson Keller, Christian M. Kramer, Wiebke Lückstädt, Christina Panknin, Tatsiana Suvorava, Brant E. Isakson, Malte Kelm, Miriam M. Cortese-Krott

**Affiliations:** ^1^ Cardiovascular Research Laboratory, Division of Cardiology, Pneumology, and Vascular Medicine, Medical Faculty, Heinrich Heine University of Düsseldorf, Düsseldorf, Germany.; ^2^ Department of Molecular Physiology and Biological Physics, Robert M. Berne Cardiovascular Research Center, University of Virginia School of Medicine, Charlottesville, Virginia.

**Keywords:** red blood cells, nitric oxide, anemia, RBC deformability, hemolysis, cardiovascular disease, red cell eNOS

## Abstract

***Significance:*** Recent clinical evidence identified anemia to be correlated with severe complications of cardiovascular disease (CVD) such as bleeding, thromboembolic events, stroke, hypertension, arrhythmias, and inflammation, particularly in elderly patients. The underlying mechanisms of these complications are largely unidentified.

***Recent Advances:*** Previously, red blood cells (RBCs) were considered exclusively as transporters of oxygen and nutrients to the tissues. More recent experimental evidence indicates that RBCs are important interorgan communication systems with additional functions, including participation in control of systemic nitric oxide metabolism, redox regulation, blood rheology, and viscosity. In this article, we aim to revise and discuss the potential impact of these noncanonical functions of RBCs and their dysfunction in the cardiovascular system and in anemia.

***Critical Issues:*** The mechanistic links between changes of RBC functional properties and cardiovascular complications related to anemia have not been untangled so far.

***Future Directions:*** To allow a better understanding of the complications associated with anemia in CVD, basic and translational science studies should be focused on identifying the role of noncanonical functions of RBCs in the cardiovascular system and on defining intrinsic and/or systemic dysfunction of RBCs in anemia and its relationship to CVD both in animal models and clinical settings. *Antioxid. Redox Signal*. 26, 718–742.

## Introduction

The main physiological role of red blood cells (RBCs), or erythrocytes is to transport of gases (O_2_, CO_2_) from the lung to the tissues and to maintain systemic acid/base equilibria. In addition, RBCs are well equipped with antioxidant systems, which essentially contribute to their function and integrity. Damage of red cell integrity, defined as hemolysis, has been shown to significantly contribute to severe pathologies, including endothelial dysfunction. Recent clinical and experimental evidence indicates that RBCs may be directly involved in tissue protection and regulation of cardiovascular homeostasis by exerting further noncanonical functions, including nitric oxide (NO) metabolism and control of blood rheology, as well as erythrocrine function (*i.e*., by releasing bioactive molecules, including NO, NO metabolites, and ATP). Many hypotheses on the role of noncanonical functions of RBCs in cardiovascular homeostasis have been put forward, and evidence of a central role played by RBCs in cardiovascular protection is accumulating. However, many aspects of RBC-mediated control of NO metabolism and ATP release are still speculative or not universally accepted.

Anemia is a pathological condition characterized by a decreased number of circulating RBCs and defined by hemoglobin (Hb) concentrations in whole blood below 12 g/dL in females and 13 g/dL in males (192). There is clinical evidence that anemia is also associated with a series of severe complications in cardiovascular disease (CVD) such as thromboembolic events (*e.g*., venous thrombosis and stroke). However, therapeutic interventions aimed to increase the circulating number of RBCs (*e.g*., by transfusion of blood or by administration of erythropoiesis-stimulating agents [ESAs] to stimulate the production of RBCs by the bone marrow), were not always effective in the tested cohorts (48, 91, 156). One possible explanation is that these treatments have side effects and therefore may contribute themselves to the negative outcome, for example, treatment with ESAs was associated with increased thromboembolic events (45).

Interpretation of large cohort studies may be very complex and requires recognition of many interacting features of disease and normal physiology. This is particularly true for studies evaluating the relationship between anemia and cardiovascular complications, which may involve different aspects, including changes in number or function of RBCs, in blood rheological properties, in systemic hemodynamics, and overall cardiovascular physiology and pathology.

**FIG. 1.**
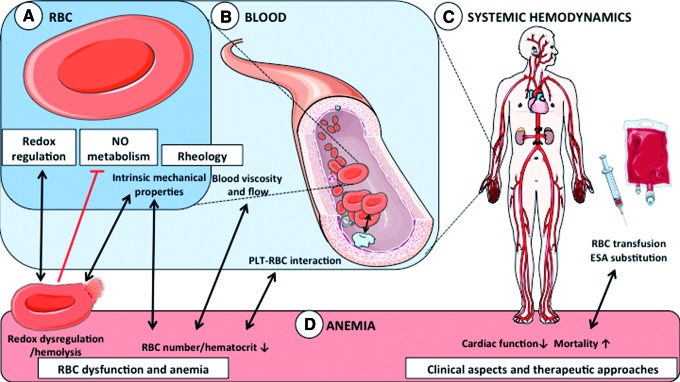
**RBC function and dysfunction: redox regulation, NO metabolism, and anemia. (A)** Intrinsic RBC properties and function. Beside their canonical role in transport of gases and nutrients, RBCs are well equipped with redox buffer systems and are important modulators of NO metabolism. Their intrinsic mechanical properties allow them to deform/change their shape in response to changes in flow and to changes in vessel diameter, thus participating in control of blood rheology. **(B)** Effects of RBCs in blood. A second way for RBCs to control blood rheology is *via* their concentration (hematocrit), which critically defines blood viscosity and blood rheology. In addition, RBCs interact with PLTs resulting in a complex cell–cell communication involving membrane adhesion molecules, NO metabolism, and redox regulation. **(C)** Effects on systemic hemodynamics. In addition to control of vascular tone and cardiac function, intrinsic RBC properties and overall blood rheology are contributors to systemic vascular hemodynamics. **(D)** Anemia. RBC dysfunction mainly results in a number of anemic conditions, which are characterized by a decrease in blood Hb concentration and circulating number of RBCs. Redox dysregulation results mainly in hemolytic anemia and release of Hb, affecting redox metabolism and NO scavenging. Anemia affects systemic hemodynamics and myocardial performance. Furthermore, patients with CVD show disturbances in hemostasis and thromboembolism and increased mortality, which cannot be effectively treated by blood transfusion or substitution of ESAs. CVD, cardiovascular disease; ESA, erythropoiesis-stimulating agent; Hb, hemoglobin; NO, nitric oxide; PLT, platelet; RBC, red blood cell. To see this illustration in color, the reader is referred to the web version of this article at www.liebertpub.com/ars

In this article, we aim to provide a chemical, biophysical, and clinical perspective about the role of RBCs in the cardiovascular system, with focus on noncanonical functions of RBCs (Fig. 1). Specifically, we will describe (I) the role of redox regulation in RBCs to maintain cell functionality and integrity, including sources of reactive oxygen species (ROS), enzymatic and nonenzymatic antioxidant systems, and damage caused by dysregulation of the redox state; (II) the complex role of RBCs in NO metabolism; (III) the intrinsic mechanical properties of RBCs and their effects on blood rheology and hemodynamics; (IV) the pathophysiology of specific anemic conditions, characterized by RBC dysfunction and hemolysis, and present mice models applied for basic and translational science studies; and (V) the clinical aspects and therapeutic approaches for anemia in CVD, outlining the open questions and proposing possible research directions.

## RBCs and Redox Regulation

The main function of RBCs is to transport oxygen from the lungs to the tissues, where it is used as a source of electrons and ATP synthesis in the mitochondria. Additionally, RBCs transport carbon dioxide (CO_2_), which is produced as a result of catabolic processes within the tissues, from the periphery to the lungs to be exhaled. CO_2_ may be transported in RBCs by Hb through reaction of amino groups of the Hb chains and formation of carbaminohemoglobin. However, most CO_2_ in the circulation is transported as bicarbonate ions (HCO_3_^−^) upon the carbonic anhydrase catalyzed reaction of CO_2_ with H_2_O, followed by H_2_CO_3_ deprotonation in water. These functions are intimately interconnected to each other: O_2_ binding affinity to the ferrous heme (Fe^2+^) of Hb is regulated by oxygen partial pressure (pO_2_), acid/base equilibria (pH), and by the levels of 2,3-diphosphoglycerate; on the other hand, CO_2_ transport is dependent on the activity of carbonic anhydrase and is directly involved in control of pH and buffering capacity of RBCs. If the ferrous heme (Fe^2+^) iron contained in the prosthetic group of Hb is oxidized to ferric (Fe^3+^) heme to form methemoglobin (metHb), the affinity of the protein toward oxygen is dramatically decreased. To preserve its functionality, Hb (which is also the most abundant cytoplasmic protein in RBCs) has to be maintained in the reduced state. The three main challenges herein are the following: first, RBCs contain numerous sources of oxidants (including high levels of molecular O_2_ bound to Hb) (89); second, RBCs carry high levels of iron within the prosthetic group of Hb (89), which in its free soluble form is a potent catalyst of ROS production *via* the Fenton reaction; and third, RBCs have limited capacity to restore damaged elements due to loss of protein expression during erythropoietic maturation.

**FIG. 2.**
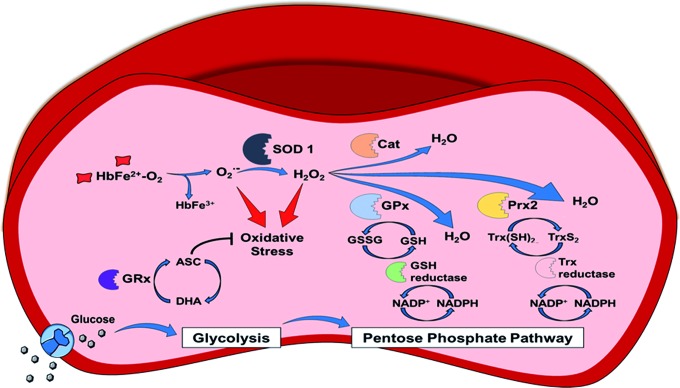
**Redox regulation in RBCs.** The occurring steady origin of ROS is shown by heme oxidation of oxyHb to metHb and the release of superoxide anions (autoxidation of Hb). The enzyme, SOD1, transforms the superoxide anions to hydrogen peroxide. Three main detoxyfication pathways exist in RBC, with GPx and Prx2 (but not Cat) pathways depending on reduced NADPH, which is synthesized by glucose uptake. Glucose is taken up mediated by the Glut-1 transporter and then gated to the glycolytic pathway. As a detour of the glycolytic pathway, glucose-6-phosphate is partly channeled into the pentose phosphate pathway producing the reduced form of NADP^+^. NADPH/glutathione (GSH)-dependent pathway: NADPH is needed as a substrate for glutathione reductase (GSH reductase) to recycle oxidized dimerized GSH (GSSG) back to reduced GSH. GSH itself is utilized by two enzymes: first, GPx for the direct breakdown of hydrogen peroxide, and second, GRx to reverse the consumption of ASC to DHA by the plasma membrane redox system and diffusing ROS. NADPH/Trx-dependent pathway: NADPH is also needed as a substrate by the Trx reductase, which keeps the cofactor Trx in the reduced state [Trx(SH)_2_]. Trx(SH)_2_ serves as an electron-delivering system to the membrane-associated Prx2 and thiols within the active center are oxidized during this process forming disulfide bridges (TrxS_2_). NADPH-independent pathway: Hydrogen peroxide breakdown by pathway 3 is independent of NADPH and catalyzation occurs by Cat. ASC, ascorbic acid; Cat, catalase; DHA, dehydroascorbic acid; GPx, glutathione peroxidase; GRx, glutaredoxin; GSSG, glutathione disulfide; metHb, methemoglobin; NADPH, nicotinamide adenine dinucleotide phosphate; Prx2, peroxiredoxin 2; ROS, reactive oxygen species; Trx, thioredoxin. To see this illustration in color, the reader is referred to the web version of this article at www.liebertpub.com/ars

In the following section, we summarize (i) the sources of oxidants in healthy RBCs, (ii) the antioxidant systems, including (ii.a) antioxidant molecules and their redox couples, such as reduced and oxidized glutathione (GSH/GSSG), ascorbate/dehydroascorbate (vitamin C), and α-tocopherol (vitamin E), (ii.b) the sources of reducing equivalents such as nicotinamide adenine dinucleotide (NADH) and nicotinamide adenine dinucleotide phosphate (NADPH), and (ii.c) the antioxidant enzymes such as superoxide dismutase (SOD), catalase (Cat), glutathione peroxidase (GPx), and peroxiredoxin 2 (Prx2); as well as (iii) the damage caused by redox dysregulation in RBCs caused by oxidants (Fig. 2).

### Sources of oxidants in RBCs

Every 24 h, 3% of Hb undergoes autoxidation, producing metHb and superoxide radical anion (Eq. 1) (50). Considering the abundance of Hb in RBCs (corresponding to 10 m*M* heme), the reaction of autoxidation of Hb is therefore the most abundant source of ROS in RBCs.

\begin{align*}
{\rm{HbF}}{\rm{e}}^{2 + }{\rm{O}}_2 \to {\rm{HbF}}{\rm{e}}^{3 +}+{\rm{O}_2}^{\bullet-}\quad\quad\quad [ 1 ]
\end{align*}

However, only 1% of Hb is present in the metHb (Fe^3+^) state. Hb-Fe^3+^ can be reduced back to Hb-Fe^2+^ in a reaction catalyzed by the cytochrome b5 reductase using NADH as an electron donor. Mutation of the gene for cytochrome b5 reductase causes cyanosis and severe neurological problems due to impairment of neuron myelination and increased mortality during childhood.

Another source of oxidants is free iron (Fe^3+^), which may dissociate from metHb (26). In the presence of free iron, the prominent reaction ongoing is the Haber–Weiss reaction (Eqs. 2 and 3), which includes the Fenton reaction (Eq. 3) producing hydroxyl radicals.

\begin{align*}
{ \rm{F}}{{ \rm{e}}^{3 + }} + {{ \rm{O}}_2}^{\bullet-}\to { \rm{F}}{{ \rm{e}}^{2 +}} + {{ \rm{O}}_2} \quad\quad\quad [ 2 ]
\end{align*}

\begin{align*}
{ \rm{F}}{ \rm{e}}^{2 + } + {{\rm{H}}_2}{{ \rm{O}}_2} \to { \rm{F}}{{ \rm{e}}^{3 + }}{ + ^ \bullet }{ \rm{OH }} + { \rm{ O}}{{ \rm{H}}^ - } \quad\quad\quad [ 3 ]
\end{align*}

The presence of ferritin in RBCs will contribute to scavenge free iron and therefore to limit the occurrence of the Haber–Weiss reaction in RBCs.

Another important oxidative pathway is the reaction between H_2_O_2_ and both ferrous and ferric Hb, resulting in heme degradation and release of free iron (3), as described in detail in an older comprehensive review by Reeder (150). These reactions lead to generation of the potent oxidizing ferrylHb species as well as secondary radicals from the reaction between H_2_O_2_ and either oxyHb or metHb. This pathway has also been proposed to be important in mediating hemolytic injury (24). Along with autoxidation (50), another important oxidative pathway is the reaction between H_2_O_2_ and both ferrous and ferric Hb, resulting in heme degradation and release of free iron (3), as described in detail before (150).

\begin{align*}
{ \rm{Hb}} - { \rm{F}}{{ \rm{e}}^{2 + }} + {{ \rm{H}}_2}{{ \rm{O}}_2} \to { \rm{Hb}} - { \rm{F}}{{ \rm{e}}^{4 + }} = {{ \rm{O}}^2}^ - + {{ \rm{H}}_2}{ \rm{O}} \quad\quad\quad [ 4 ]
\end{align*}

\begin{align*}
{ \rm{Hb}} - { \rm{F}}{{ \rm{e}}^{3 + }} + {{ \rm{H}}_2}{{ \rm{O}}_2} \to { \rm{H}}{{ \rm{b}}^{ \bullet + }} - { \rm{F}}{{ \rm{e}}^{4 + }} = {{ \rm{O}}^2}^ - + {{ \rm{H}}_2}{ \rm{O}} \quad\quad\quad [ 5 ]
\end{align*}

\begin{align*}
{ \rm{Hb}} - { \rm{F}}{{ \rm{e}}^{4 + }} = {{ \rm{O}}^2}^ - + {{ \rm{H}}_2}{{ \rm{O}}_2} \to { \rm{Hb}} - { \rm{F}}{{ \rm{e}}^{3 + }} + {{ \rm{O}}_2}^{\bullet-} + {{ \rm{H}}_2}{ \rm{O}} \quad\quad\quad [ 6 ]
\end{align*}

\begin{align*}
{ \rm{Hb}} - { \rm{F}}{{ \rm{e}}^{3 + }} + {{ \rm{O}}_2}^{\bullet-} \to { \rm{heme \ degradation \ products}} + { \rm{F}}{{ \rm{e}}^{3 + }} \quad\quad\quad [ 7 ]
\end{align*}

As described in more detail in the RBCs and NO Metabolism section, RBCs are thought to generate measurable levels of NO. Therefore, taking into account the almost diffusion-limited rate constant for the reaction between NO and O_2_^•−^, it would seem at least reasonable that peroxynitrite could be formed according to Eq. 8.

\begin{align*}
{ \rm{NO}} + {{ \rm{O}}_2}^{\bullet-} \to { \rm{ONO}}{{ \rm{O}}^ - } \quad\quad\quad [ 8 ]
\end{align*}

It is important to note that antioxidant systems abundant in RBCs, including Prx and GPx, as well as metHb are also potent peroxynitrite scavengers.

### Antioxidant systems

The antioxidant systems of RBCs are based on both enzymatic and nonenzymatic mechanisms. RBCs carry (I) high concentrations of antioxidant molecules, including glutathione (GSH) and vitamins such as ascorbic acid (vitamin C) and α-tocopherol (vitamin E), (II) sources of reduced equivalents such as NADH and NADPH, and (III) antioxidant enzymes, including SOD1, Cat, GPx, and the thioredoxin (Trx) system, as described in detail below. The antioxidant systems in RBCs strongly contribute to keep the levels of oxidants (including O_2_^•−^, H_2_O_2_ and other reactive species described in the section “Sources of oxidants in RBCs”) very low.

#### I. Antioxidant molecules and redox couples

##### GSH/GSSG redox couple

Antioxidant molecules abundant in RBCs are the GSH/GSSG redox couple, ascorbate and alpha-tocopherol. GSH is a tripeptide consisting of L-glutamine, L-cysteine, and L-glycine synthesized by a γ-glutamine-cysteine ligase and a glutathione synthase. In healthy human RBCs, 90%–95% of glutathione is present in the reduced form GSH (200) that can be utilized for the reduction of ascorbate, oxidized proteins, and oxidized lipids (184). Enzymes using GSH as reducing equivalent are glutaredoxin (GRx) and GPx. The enzyme responsible for GSH recycling is glutathione reductase, which reduces glutathione disulfide (GSSG) back to the reduced GSH *via* consumption of NADPH (197).

##### Ascorbate/dehydroascorbate

Ascorbate is an essential hydrophilic vitamin and an important reducing equivalent in RBCs, which is (re)synthesized from dehydroascorbic acid at the expense of GSH in a reaction catalyzed by a variety of enzymes, especially GRxs (190), which are small cytoplasmic enzymes also catalyzing deglutathionylation, reduction of protein disulfides, or Fe-S linkage formation. The major transport mechanism of ascorbate into the RBC is attributed to glucose transporter 1 (116, 130). It is involved in several antioxidant mechanisms such as reduction of diffusible oxidants and metHb, as well as maintenance of plasma membrane redox system (PMRS) (51). The PMRS is a system transferring electrons from the intracellular cytosolic RBC compartment to the extracellular medium (plasma) by oxidation of intracellular electron donors (*e.g*., ascorbate and NADH), thus driving the exchange of electrons and keeping plasma components in a reduced state. The physiological significance of the PMRS is not completely understood, but it was reported that the increased PMRS activity in erythrocytes during aging may be a protective mechanism of the system for efficient extracellular dehydroascorbate reduction and ascorbate recycling under conditions of increased oxidative stress (173). Thus, PMRS in RBCs may act as a compensatory mechanism against increased ROS/oxidative stress and represents a potential mechanism of how RBCs contribute to redox regulation throughout the whole body (173).

##### α-Tocopherol (vitamin E)

α-Tocopherol is an important oxidant scavenger in RBCs. Due to its lipophilicity, α-tocopherol accumulates in RBC membranes and plays a central role in preventing lipid peroxidation, probably by limiting the amplification of peroxidation chain reactions within the plasma membrane (24). It was demonstrated that recycling of α-tocopherol radicals, next to diffusion of new molecules out of plasma, occurs by the oxidation of ascorbate (24, 121).

#### II. Sources of redox equivalents (NADH and NADPH)

Like other cell types, both NADH and NADPH are sources of reducing equivalents in RBCs. In contrast to other cells, the pentose phosphate cycle is the main source of reducing equivalents in the RBCs due to a lack of mitochondria. Glucose-6-phosphate dehydrogenase (G6PDH) retrieves glucose-6-phosphate from glycolytic ATP production and starts NADP^+^ reduction. Interestingly, the antioxidant defense is tightly connected to the energy status of the RBC. Excessive fasting can lead to decreased levels of reduced NADPH in RBCs caused by decreased availability of glucose to the glycolytic pathway. In addition, the lack of riboflavins—components of the cosubstrate flavin adenine dinucleotide (FAD) important for glutathione reductase functionality—can impair antioxidant defense in fasting (10). NADPH is of high importance for redox balance within the RBC. Malfunction of enzymes within the pentose phosphate pathway can have severe consequences on overall membrane stability and permeability, as will be discussed in the section “Redox Dysregulation in RBCs and Hemolytic Anemia”.

#### III. Enzymatic antioxidant systems (SOD, Cat, GPx, Prx2)

The enzymes known to participate in the processing of oxidants in mature RBCs are SOD1, Cat, GPx, and Prx2 (129). The family of SOD enzymes comprises three isoforms with different structural characteristics of the prosthetic group (containing manganese or copper and zinc) as well as compartmentalization and functional significance (118). Of high importance for the mature RBC is the copper/zinc isoform 1 of SODs (SOD1), catalyzing the dismutation of the superoxide anion (Eq. 9), which is formed in RBCs mostly by Hb autoxidation (Eq. 1), into hydrogen peroxide (122).

\begin{align*}
2{{ \rm{O}}_2}^{\bullet-} + 2 \ {{ \rm{H}}^ + } \to {{ \rm{H}}_2}{{ \rm{O}}_2} + {{ \rm{O}}_2} \quad\quad\quad [ 9 ]
\end{align*}

Hydrogen peroxide hereupon is degraded to oxygen and water by Cat, GPx, or Prx2. In nonpathological conditions, GPx has been shown to be the enzyme degrading most of the H_2_O_2_ by oxidizing reduced GSH into GSSG (Eq. 10). GSSG is recycled by GSH reductase, which consumes NAPDH (38). In conditions of overproduction of O_2_^•−^ and therefore higher concentrations of H_2_O_2_, Cat takes over and reduces H_2_O_2_ into water (Eqs. 11 and 12) (89).

\begin{align*}
{{ \rm{H}}_2}{{ \rm{O}}_2} + 2 \ { \rm{ GSH}} \to 2 \ {{ \rm{H}}_2}{ \rm{O}} + { \rm{GSSG}} \quad\quad\quad [ 10 ]
\end{align*}

\begin{align*}
{ \rm{catalase }} \ \left( {{ \rm{F}}{{ \rm{e}}^{3 + }}} \right) + {{ \rm{H}}_2}{{ \rm{O}}_2} \to { \rm{compound \ I}} + {{ \rm{H}}_2}{ \rm{O}} \quad\quad\quad [ 11 ]
\end{align*}

\begin{align*}
{ \rm{compound \ I}} + {{ \rm{H}}_2}{{ \rm{O}}_2} \to { \rm{catalase}} + {{ \rm{O}}_2} + {{ \rm{H}}_2}{ \rm{O}} \quad\quad\quad [ 12 ]
\end{align*}

Although traditional descriptions of RBCs postulate the lack of intracellular compartmentalization of enzymes due to a lack of nuclei and organelles (including mitochondria), subcellular localization of these antioxidant systems plays a fundamental role. The association of Prx2 (which participates in reduction of organic peroxides, Eq. 13) with the membrane (120) makes it important for protection of RBC membrane constituents against lipid peroxidation. A lack of this enzyme has greater impact on the phenotype than lack of GPx or Cat (106).

Prx2 retrieves its electrons for hydrogen peroxide reduction from the reducing equivalent Trx (Eq. 14), which is dependent on a sufficient supply of NADPH.

\begin{align*}
{ \rm{Prx}} ( {{ \rm{S}}^ - } ) + {{ \rm{H}}_2}{{ \rm{O}}_2} \to { \rm{Prx}} ( { \rm{S}}{{ \rm{O}}^ - } ) + {{ \rm{H}}_2}{ \rm{O}} \quad\quad\quad [ 13 ]
\end{align*}

\begin{align*}
{ \rm{Prx}} ( { \rm{S}}{{ \rm{O}}^ - } ) + { \rm{Trx}}{ \left( {{ \rm{SH}}} \right) _2} \to { \rm{Prx}} ( {{ \rm{S}}^ - } ) + { \rm{Trx}} \left( {{{ \rm{S}}_2}} \right) + {{ \rm{H}}_2}{ \rm{O}} \quad\quad\quad [ 14 ]
\end{align*}

### Damage caused by oxidation reactions and redox dysregulation

The coordinated action of all antioxidant systems makes RBCs very resistant against oxidation as well as an efficient systemic redox buffering system. Malfunction of antioxidant defense and/or conditions of increased oxidant production have severe consequences for RBCs on a subcellular level. These consequences include degradation of Hb (Eqs. 4–7) and other proteins, disturbance of ionic homeostasis (Ca^2+^) (49), formation of neoantigens (96), hindered RBC deformation (37), interference with erythropoiesis (162), and enhanced exposure of phosphatidylserine (PS) (85). High concentrations of polyunsaturated fatty acids in RBC membrane make them susceptible to peroxidation leading to the loss of RBC membrane integrity and decreased activity of membrane enzymes [*e.g*., ATPase (198) and acetylcholinesterase (183)].

### Biochemical consequences of hemolysis

Hemolysis in the circulation has been shown to exert profound pathological effects, particularly on the cardiovascular system. The consequence of hemolysis is particularly evident in hemolytic anemia, where fragility of RBCs is mainly due to redox dysbalance or hemoglobinopathies, for example, sickle cell disease (SCD) (see the section “RBC Dysfunction and Anemia” for more details), and observed in transfusion of older blood (71). The main consequence of RBC rupture is the release of intracellular content, in particular Hb and arginase 1. Loss of Hb compartmentalization may lead to systemic NO scavenging and may affect endothelial function (72, 153). According to Eqs. 4–7, Hb may also react with H_2_O_2_ (*e.g*., generated during inflammatory conditions), promote ferrylHb formation, and induce lipid peroxidation (3). Hb or heme has been proposed to activate Toll-like receptor 4 and induce proinflammatory nuclear factor kappa-light-chain-enhancer of activated B cell-dependent signaling (16, 67). Moreover, release of arginase 1 from the RBCs was proposed to contribute to arginine depletion, leading to decreased endothelial nitric oxide synthase (eNOS) activity (131).

### Summary: redox buffering function of RBCs

To summarize, RBCs are particularly well equipped with potent nonenzymatic and enzymatic antioxidant systems, which are important to maintain Hb in a reduced oxygen binding form, to limit oxidative modifications of membrane lipids, structural proteins, channels, and metabolic enzymes, and therefore to keep the cell alive and functional for its (average) 120-day life. Failure or dysfunction of the antioxidant system may have severe consequences for the cell, including loss of membrane integrity leading to hemolytic anemia. Beyond this, RBC antioxidant pathways and their ability to reduce extracellular antioxidants *via* the transmembrane electron transport system, along with RBC mobility through the circulation, make them an ideal ROS buffering component, which may contribute to overall systemic homeostasis of redox balance.

## RBCs and NO Metabolism

RBCs were considered for a long time as a powerful scavenger of endothelial cell-derived NO, participating in systemic NO metabolism mainly by limiting NO bioavailability. Recent evidence indicates that RBCs participate in systemic NO metabolism and regulation of vascular tone and integrity, but in a different way than initially thought.

NO is constitutively produced within the endothelium by eNOS and it is thought to freely diffuse into smooth muscle cells, where NO binds to the Fe^2+^-heme center of a soluble guanylyl cyclase leading to vasodilation. Hb is thought to rapidly inactivate the NO signal by binding NO to the oxygenated Fe^2+^-heme metal center, which is followed by subsequent formation of metHb and nitrate with a high reaction constant of ≈6–8 × 10^7^ *M*^−1^s^−1^ (Eq. 15) (81). Moreover, under hypoxic conditions, NO can also rapidly react with deoxygenated Hb, leading to formation of nitrosylhemoglobin (Eq. 16):

\begin{align*}
{ \rm{HbF}}{{ \rm{e}}^{2 + }}{{ \rm{O}}_2} + { \rm{NO}} \to { \rm{HbF}}{{ \rm{e}}^{3 + }} + { \rm{N}}{{ \rm{O}}_3}^ - \quad\quad\quad [ 15 ]
\end{align*}

\begin{align*}
{ \rm{HbF}}{{ \rm{e}}^{2 + }} + { \rm{NO}} \to { \rm{HbFe}} - { \rm{NO}} \quad\quad\quad [ 16 ]
\end{align*}

Since RBCs contain 10 m*M* Hb (corresponding to 97% of erythrocytes' dry weight) (81), they were thought to represent an irreversible sink of endothelium-derived NO under any conditions. Interestingly, the nature of NO as an endothelial-derived relaxing factor was initially not universally accepted because of these reactions: the reasoning of many researchers in this field was that the presence of a highly efficient scavenger in direct vicinity of the main source of NO would produce a concentration gradient leading to diffusion of NO into the blood stream (instead of into the smooth muscle) and thereby prevent vasodilatory function. However, *in vitro* experiments have shown that there is a 1000-fold discrepancy between the reaction with free Hb or intact RBCs (111). Thanks to its small size and lack of charge, NO can easily diffuse through plasma membranes, but its diffusion into the RBCs is limited by transmembrane and intracellular resistances (185), as well as by the presence of an unstirred layer of solvent around the RBC (extracellular resistance) (46). This may explain lower consumption of NO by RBCs compared with free Hb.

This view changed radically after the discovery of RBC-mediated hypoxic vasodilation: under conditions of decreased oxygen saturation, RBCs were shown to induce vasodilation of vessel strips by exporting NO bioactivity (146). Formation of nitrosothiols, and in particular S-nitrosohemoglobin, was proposed to be the signal mediating hypoxic vasodilation. In contrast, another study demonstrated that Cys-93 in the β-subunit of Hb (where this nitrosothiol is formed) is not essential for RBC-dependent hypoxic vasodilation (83), thus implicating other NO metabolites or pathways. The observation that RBCs contain high concentrations of nitrite (44) in an arterial-venous gradient (72) and that deoxyhemoglobin catalyzes nitrite reduction started a lively debate about the origin of NO released from RBCs—whether by formation of S-nitrosohemoglobin or nitrite reduction by Hb. The latest hypothesis is supported by the fact that RBC-mediated nitrite reduction and subsequent inhibition of platelet (PLT) aggregation do not take place with nitrite alone (2, 144).

\begin{align*}
{ \rm{HbF}}{{ \rm{e}}^{2 + }} + { \rm{N}}{{ \rm{O}}_2}^ - + {{ \rm{H}}^ + } \to { \rm{HbF}}{{ \rm{e}}^{3 + }} + { \rm{NO}} + { \rm{O}}{{ \rm{H}}^ - } \quad\quad\quad [ 17 ]
\end{align*}

Other proposed nitrite reductases catalyzing NO formation from nitrite under hypoxic conditions are carbonic anhydrase (1), xanthine oxidase (68), or eNOS (189), which are all present in RBCs. However, it was shown recently that deoxyhemoglobin may be the only nitrite reductase of physiological relevance under hypoxic conditions (Eq. 17) as inactivation of deoxyhemoglobin by treatment with carbon monoxide completely blocked NO release from RBCs as assessed by electron paramagnetic resonance (107).

The main controversial issue of all the studies proposing that NO is released from RBCs is understanding how exactly NO can escape scavenging by Hb in the red cell, can be released, and can reach its targets. It was proposed that HbNO oxidation by nitrogen dioxide could help release NO and minimize the effects of autocapture (75). In addition, Gladwin and Lancaster each independently proposed a slight variation of a nitrite anhydrase mechanism in which the product of nitrite reduction is N_2_O_3_ (72, 111). This reaction cycle could, in principle, facilitate NO escape from the RBC due to the slightly longer half-life of N_2_O_3_, which could then homolyze to NO and NO_2_ outside the cell according to Eq. 18.

\begin{align*}
{{ \rm{N}}_2}{{ \rm{O}}_3}{ \to ^ \bullet }{ \rm{NO}}{ + ^ \bullet }{ \rm{N}}{{ \rm{O}}_2} \quad\quad\quad [ 18 ]
\end{align*}

However, these mechanisms are not universally accepted, as pointed out in a recent work by Koppenol (101). A recent study proposed that only 25% of nitrite is directly reduced into NO, while the rest of the nitrite forms two metastable intermediates of [Fe^2+^Hb···NO_2_^−^] and [Fe^3+^Hb-NO ↔ Fe^2+^Hb-NO^+^], which holds a >100-fold affinity for the membrane. The proximity of these species to the RBC membrane makes NO release and export possible, which should occur after conformational changes of Hb induced by the presence of the membrane (157).

**FIG. 3.**
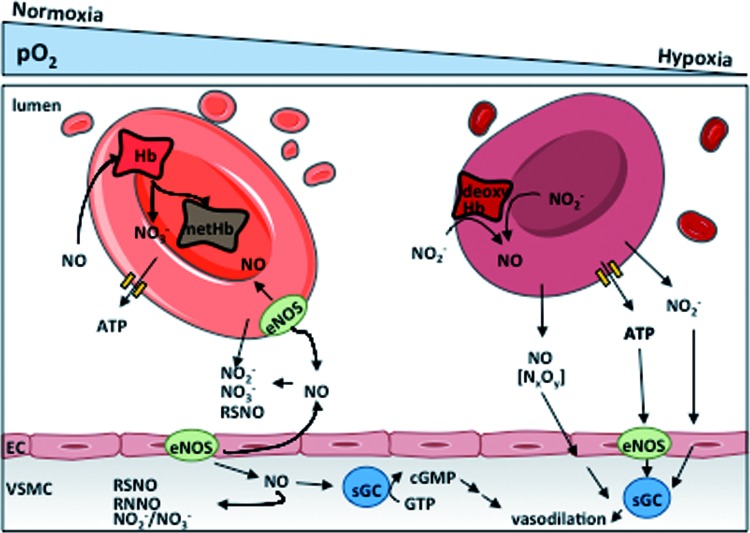
**Role of RBCs in systemic NO metabolism**. NO is a vasodilator produced by eNOS enzymes in ECs and mediates vasodilation by activating the soluble guanylate cylase in VSMCs. NO is inactivated by RBCs by reaction with oxyHb forming metHb and nitrate. In the plasma, tissues and RBCs, NO can be oxidized to NO_2_^−^ or NO_3_^−^ and to other metabolites. Under hypoxic conditions, NO_2_^−^ can react with deoxyHb to form NO, which was proposed to mediate hypoxic vasodilation and to inhibit platelet aggregation. Additionally, hypoxic ATP release by RBCs was also proposed to induce vasodilation by activating eNOS in the endothelium. ATP, adenosine triphosphate; ECs, endothelial cells; eNOS, endothelial nitric oxide synthase; sGC, soluble guanylate cyclase; VSMC, vascular smooth muscle cell. To see this illustration in color, the reader is referred to the web version of this article at www.liebertpub.com/ars

Another mechanism proposed to participate in RBC-mediated hypoxic vasodilation is the release of ATP from RBCs. Shear stress, hypoxia, or pharmacological stimuli, such as β-adrenergic receptor stimulation, have been shown to activate signaling cascades leading to release of ATP through cystic fibrosis transmembrane conductance regulator or pannexin 1 channels (36, 53). After binding to vascular endothelial purinergic receptors, ATP was proposed to induce cyclic adenosine monophosphate (cAMP)-mediated activation of a phosphorylation cascade leading to eNOS activation and subsequent vasodilation (Fig. 3). Thus, RBCs induce NO synthesis in the vessel wall and thereby participate indirectly in maintenance of NO bioavailability.

These pathways have been proposed to be of central importance for matching of oxygen demand to its delivery in the microcirculation of the skeletal muscle during hypoxic work (53). Likewise, a crosstalk of nitrite export mechanisms and ATP release in hypoxic vasodilation was also reported (29). These data were recently challenged in a methodological article by a significant correlation between ATP release and hemolysis (measured as Hb concentration in the supernatant), leading to a still ongoing discussion on the significance of RBC-mediated ATP release (98, 167).

The presence of enzymatic NO production from L-arginine (L-Arg) *via* NOS in RBCs was proposed two decades ago and controversially discussed in the literature (39). Recently, the NOS in RBCs was identified as a catalytically active eNOS (NOS3 type 1), which is identical to the eNOS expressed in endothelium, as assessed by immunoprecipitation and liquid chromatography mass spectroscopy (40), and confirmed by others through the analysis of conversion of L-^15^N-arginine into L-^15^N-citrulline (52).

The first indication of an *in vivo* relevance of blood eNOS was put forward by creation of irradiated and bone marrow transplanted mice (chimeras) that were lacking eNOS in blood; these chimera mice present a hypertensive phenotype along with decreased circulating nitrite and nitrate levels (196), as well as increased infarct sizes after myocardial ischemia/reperfusion injury (73, 125). These results show that not only nitrite-derived NO production by RBCs under hypoxic conditions but also red cell eNOS-derived NO may play a role in controlling cardiovascular hemodynamics.

A correlate with endothelium was recently described where Hb-α was found to be expressed in myoendothelial junctions (connection between endothelium and smooth muscle) in resistance vessels without its β subunit partner (172). Functionally, the endothelial alpha globin was shown to regulate NO availability *via* direct interaction with eNOS in these cells (171). Fine-tuning of NO diffusion from the endothelium to the smooth muscle cell is controlled by regulation of its Fe^2+^/Fe^3+^ redox equilibrium (172). This makes an important parallel between RBC Hb and vascular Hb, where control of redox state of Hb in both compartments is responsible for systemic control of NO bioavailability, NO metabolism, and results in fine regulation of cardiovascular hemodynamics, further underscoring the importance of NO bioavailability in both systems.

Taken together, there is accumulating evidence that RBCs play an important role in control of systemic NO metabolism, transport, and (as discussed in the RBC Mechanical Properties and Blood Rheology section) release of vasoactive substances, participating in systemic control of cardiovascular function and cardioprotection. Further research should be focused on how changes in number, structure, or function of RBCs may affect NO metabolism and cardiovascular homeostasis and may help to identify/reveal clinically relevant molecular mechanisms underpinning the correlation between anemia, bleeding complications, and severe outcome in CVD.

## RBC Mechanical Properties and Blood Rheology

Tissue oxygenation and export of CO_2_ are the main goals of the circulatory system. To fullfill this role, the circulation of blood is tightly controlled by the cardiovascular system (*i.e.*, by modulation of cardiac function and vascular tone) as well as by the biophysical properties of blood itself. Being the major cellular component of blood, RBCs play a central role in defining biophysics of blood rheology as well as efficiency of tissue perfusion and gas exchange. In this section, we will summarize (i) the intrinsic biophysical characteristics of RBCs that define their mechanical properties and their ability to deform, such as RBC geometry, intracellular viscosity, and cytoplasmic protein flexibility; (ii) how these properties significantly contribute to rheological characteristics of blood such as blood viscosity; and (iii) what is known about the biochemical pathways regulating the mechanical properties of RBCs.

### RBC deformation and mechanical properties

From the high flow conditions in the aorta to the single micron-wide capillaries, RBCs experience a large range of flow conditions and changes in shear stress and hemodynamic forces. To deliver oxygen to the tissues, erythrocytes must be able to dynamically adapt to and compensate for the constantly changing flow conditions along the vascular tree and particularly in the narrowest capillaries to deform significantly without rupturing. Assuming standard cardiovascular parameters for a human (cardiac output = 4.0–8.0 L/min, total blood volume = 5–6 L), one can calculate that a single erythrocyte traverses the circulatory system in ∼1 min; therefore, during its average lifetime of 120 days (42), one RBC undergoes on the order of 10^5^ cycles of high flow conditions in the aorta, followed by squeezing through the capillaries.

**FIG. 4.**
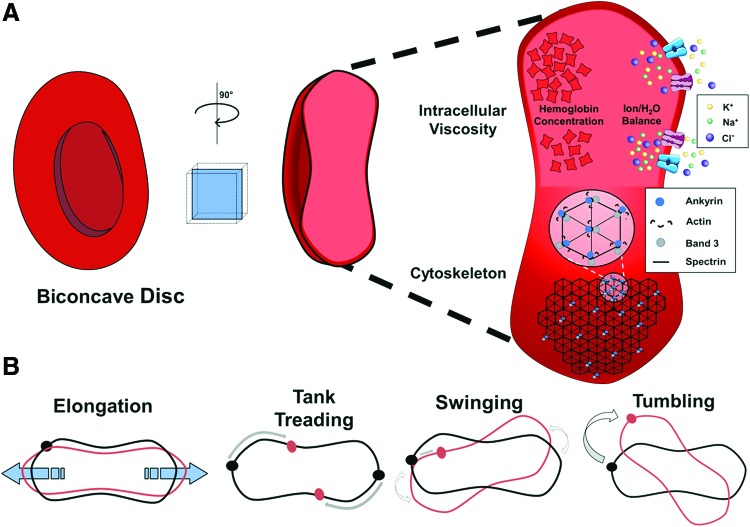
**RBC shape, deformation, and mechanical properties. (A)** RBCs can deform from the normal biconcave disc shape due to changes in flow conditions in the blood stream. Deformability of a single cell is primarily determined by viscosity of the cytoplasm and flexibility of the cytoskeleton. Intracellular viscosity is mostly due to two factors, the concentration of Hb (the most abundant protein in the RBC) and water content due to osmolarity balancing effects. The membrane shape is affected by the cytoskeleton scaffold, which is formed by spectrin links between membrane-associated protein complexes, comprising ankyrin, band 3, and actin, among other proteins. **(B)** Modes of deformation of RBCs. There are four distinct ways that RBCs can deform while traveling in the bloodstream. Elongation of the discoid shape happens in response to shear stress along the elongation axis. Membrane tanktreading is movement of the membrane around the discoid shape without significant changes in the shape of the whole cell. This movement has been shown to force the RBC into the center of the vessel, promoting blood flow. Swinging is membrane tanktreading combined with small oscillations in the direction of the principal axis of the ellipsoid shape. Finally, end-over-end tumbling of RBCs is possible and promotes turbulent flow of cells within the blood stream. To see this illustration in color, the reader is referred to the web version of this article at www.liebertpub.com/ars

Normal RBCs have a few possible modes of changing their shape (114, 168, 188). In high shear rates, *elongation* of the principal ellipsoid axis is observed. This is the classical definition of cell deformability and is measured *via* ektacytometry. Other modes of motion in response to changes in flow are also *end-over-end tumbling* and *periodic swinging* (Fig. 4), which are not changes in shape for an individual RBC, but can affect total flow conditions and viscosity of whole blood (60, 61). Additionally, *membrane tanktreading*, where points on the RBC membrane traverse around the discoid without the cell changing shape, presents another observed mode of RBC motion (Fig. 4). Tanktreading is a favorable deformation mode because it creates lift forces that push individual RBCs away from vessel walls and into the center of the blood stream, where the flow is greatest. The combination of all of these motions links individual cell properties with the overall rheology of blood.

The characteristics of RBCs allowing them to undergo the necessary deformations include (i) the overall geometry of the cell, characterized by discoid donut-like shape increasing cell flexibility and creating high cellular surface area-to-volume ratio for efficient gas exchange; (ii) the cytoplasmic viscosity determined by intracellular Hb and ionic/H_2_O concentrations defining the intrinsic resistance of the cell to external forces; and (iii) membrane/cytoskeleton flexibility and mechanical properties.

#### Geometry of the cell

The discoid donut-like shape of RBCs is fundamental for their physiological function as it increases overall cell flexibility and creates a high cellular surface area-to-volume ratio allowing for efficient gas exchange. The discoid shape is defined by cytoskeletal structural proteins and ion channels (127). To maintain a discoid shape, the internal volume of RBCs must not exceed a threshold that would overcome cytoskeleton organization forces. The average internal volume of an RBC is 94 μm^3^ at 300 mOsmol/kg. Disorders of the RBC cytoskeleton lead to changes in overall shape of the cells such as spherocytosis and elliptocytosis. The abnormal cellular morphology and decreased surface area-to-volume ratio in these disorders decrease deformability and the ability of cells to move through capillary beds and exchange gases.

#### Cytoplasmic viscosity

Early work on the fluid dynamics of RBCs determined that the form of deformation depends heavily on both the internal viscosity of the cell and the membrane viscosity. The ratio of cytoplasmic and external viscosities (often termed lambda, λ) determines the mode of deformation, with a greater deformation at lower λ values (148). Tumbling of cells *in vivo* was associated with a greater λ value in the same study. Increased λ is also associated with less tanktreading movement, although the effect of cytoplasmic viscosity is less than that of membrane viscosity.

Membrane fluidity has been implicated in RBC deformability. Increased cholesterol and phospholipid ratios in the membranes of RBCs are correlated with decreased RBC deformability (25, 123). Disruption of normal lipid composition of the outer sheath of the RBC membrane bilayer has been observed in diabetic patients and is hypothesized to contribute to impaired deformability and subsequent blood flow disruptions. Thus, cytoplasmic and membrane viscosity of RBCs may affect systemic flow of RBCs through physical indices of cellular deformability.

#### Structure and mechanical properties of the cytoskeleton

The intrinsic ability of RBCs to change their shape is mainly defined by their flexibility as well as stability of the inner membrane-attached cytoskeleton. In RBCs, cytoskeletal proteins (mainly spectrin, actin, band 3, and ankyrin) form a pseudohexagonal structure. This structure creates a dynamic scaffold that supports integral membrane proteins and promotes integrity of the cells under constant stress (115). The flexibility of the RBC membrane depends on the interactions of cytoskeletal proteins, spectrin and actin (109), as well as on the intrinsic plasticity of the spectrin molecule which is due to its peculiar macromolecular structure (104). Flexibility of the RBC is also ensured by variable oligomerization of spectrin molecules under shear-stress conditions and by melting of secondary structure of spectrin, thus allowing for a wide range of length scales of the RBC cytoskeleton (88, 110). Additionally, spectrin provides stability under a variety of shearing forces by cross-linking actin with other RBC membrane proteins (177). The equilibrium of spectrin–actin interaction along with transmembrane proteins (*e.g.*, ion channels, receptors) and membrane-associated proteins (ankyrin, band 3, *etc*.) gives a functional shape to the RBC and allows for elasticity (*i.e*., reversible deformation) of the cell. In summary, these intrinsic biophysical characteristics of RBCs allow them to survive repeated cycles of high flow in big arteries and squeezing in small-diameter capillaries.

### Rheological properties of blood

From a biophysical perspective, blood can be considered as a suspension of flexible and elastic particles (blood cells) in a viscous fluid (plasma). RBCs are the major cellular component of this cell suspension and occupy 40%–45% volume percentage (defined as hematocrit) in women and men, respectively. Using laminar flow assumptions that do not fully incorporate the properties of blood, plasma viscosity (*η*) can be considered as constant, that is, the rheological properties of the plasma obey the Newton law

\begin{align*}
\tau = \eta \times \gamma \quad\quad\quad [ 19 ]
\end{align*}

where τ is the shear stress and *γ* the shear rate.

Blood instead behaves as a shear-thinning non-Newtonian fluid during flow so that increased shear stress on blood decreases blood viscosity (*i.e*., thinning of the liquid). Although RBCs are not the only factor in blood that can cause a change in viscosity, they dominate viscosity calculations due to their high preponderance. The characteristics of RBCs that define blood viscosity are (i) the elastic deformation of RBCs in response to mechanical forces such as transitions from end-over-end tumbling to membrane tanktreading (60); (ii) the ability of RBCs to orient themselves with the flow; (iii) the tendency of RBCs to aggregate at low flow conditions and form rouleaux, *e.g.*, stacks of cells similar to a stack of coins); and (iv) the local cell concentration (*i.e*., hematocrit). While hematocrit values are the major determinant of blood viscosity, as a more packed fluid (increased hematocrit) blood is more resistant to flow (increased viscosity), a loss of deformability in RBCs may increase viscosity significantly (34) due to increased end-over-end tumbling (61). Other non-RBC-related mechanisms, but equally important parameters, which define blood viscosity, are the diameter of the vessels and systemic hemodynamics.

### Modulation of RBC deformability and blood rheology by oxidants and NO

Changes in the redox state of RBCs by treatment with alkylating agents, superoxide-generating systems, and peroxides strongly affect RBC deformability (12). Interestingly, in malaria, heme oxidation products derived from RBC lysis have deleterious effects on RBC deformability and were proposed to be responsible for changes in blood flow and microcirculation (141).

In addition, NO has been proposed as a critical regulator of cell deformability (22, 93, 181), although some controversy in the literature exists about these effects (15). Administration of NOS inhibitors decreased RBC deformability as assessed by ektacytometry (22), while low concentrations of NO donors increase RBC deformability (22), membrane fluidity (181), and RBC filterability (99). However, recently, the effects of NO donors on RBCs deformability *ex vivo* were not confirmed by others (15). In microcirculation of the chorioallantoic membrane of the chicken egg, eNOS inhibition and NO donors affected RBC deformation and velocity independently from changes of the vascular diameter (82) and administration of L-arginine normalized deformability.

One important activator of production of NO in the endothelium is shear stress (43, 163). It was reported that a small increase in plasma viscosity (whether by increased hematocrit or non-O_2_-carrying dextran molecules) increases NO release by endothelium and can lead to decreased blood pressure (27, 119, 179). On the other hand, it was shown that a decreased hematocrit due to iron deficiency anemia may decrease RBC-mediated NO scavenging, leading to hypotension (140). The complex relationship between modulation of RBC deformability by NO and oxidants, and their effects on viscosity and vascular shear stress should be investigated in more detail.

### Summary: functional significance of RBC mechanical properties

RBC structural and biophysical characteristics are optimized for canonical functions of RBCs such as gas transport and exchange to the tissues. To accomplish this, RBCs need to dynamically adapt to and compensate for the constantly changing flow conditions along the vascular tree, as well as to squeeze, deform, and pass through narrow capillaries without rupture and obstructing the vessels. These intrinsic elastic properties of RBCs depend on structural and biophysical characteristics, including cell geometry, intracellular viscosity, and flexibility of the cytoskeleton. These RBC intrinsic properties, together with the mechanisms regulating vascular tone and cardiac function, contribute to regulation of blood rheology, peripheral resistance, and cardiac function (114, 168, 188). Further research should address the question how these biophysical intrinsic RBC properties are affected by changes in biochemical pathways regulating RBC redox state and NO metabolism.

## RBC Dysfunction and Anemia

**FIG. 5.**
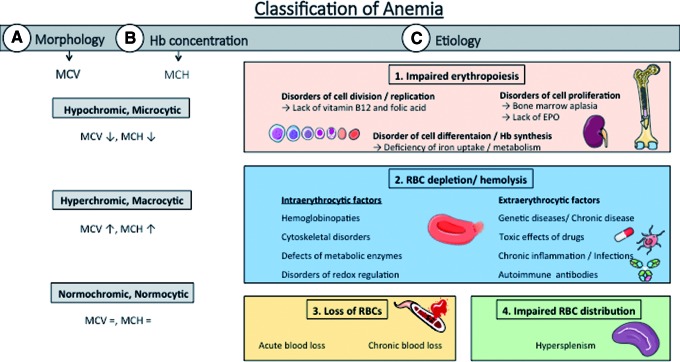
**Classification of Anemia.** Anemic conditions can be classified according to their **(A)** morphology/size of the circulating RBCs, *i.e.*, normocytic, macrocytic, and microcytic anemia, assessed as the mean corpuscular volume (MCV); **(B)** the concentration of Hb in RBCs, *i.e.*, normochromic, hypochromic, and hyperchromic anemia, mainly determined by MCH; or **(C)** etiopathology, *i.e.*, due to **(1.)** defects in erythropoiesis (*i.e*., due to a lack of iron or EPO); **(2.)** increased hemolysis or RBC degradation in the circulation (which may have a number of different intra- or extraerythrocytic reasons); **(3.)** acute or chronic bleeding; or **(4.)** a disorder of cell distribution with an increased RBC uptake by the reticuloendothelial system of the spleen. The cytological characteristics of anemia may correspond to multiple etiologies. For example, hypochromic microcytic anemia can be caused by either lack of iron or increased hemolysis or chronic blood loss. EPO, erythropoietin; MCH, mean corpuscular hemoglobin; MCV, mean corpuscular volume. To see this illustration in color, the reader is referred to the web version of this article at www.liebertpub.com/ars

According to the World Health Organization, anemia is defined as a pathological condition characterized by Hb concentration in whole blood below 12 g/dL in females and 13 g/dL in males (192) and by a decreased number in circulating RBCs. There are different forms of anemia, which are mainly classified according to changes in morphology of RBCs, concentration of Hb, and etiopathology (Fig. 5).

In this section, we focus on how dysfunction of intrinsic homeostatic mechanisms in RBCs may lead to anemic conditions both in humans and specific mouse models. In particular, we will (I) introduce a general classification of anemic conditions; (II) describe types of anemia induced by RBC dysfunction, including (II.a) defects of Hb chain compositions (defined as hemoglobinopathies) such as SCD and thalassemia; (II.b) defects of cytoskeletal proteins; and (II.c) redox dysregulation and hemolytic anemia. In Table 1, a list of relevant animal models of anemia is provided, which may allow untangling the molecular mechanisms of RBC dysfunction leading to anemia.

**Table 1.** Etiological Classification of Anemia and Characterized Mice Models

Disorder of erythropoiesis	Iron deficiency	Diet
		Genetical: Trf(hpx/hpx) gene mutation (hypotransferrinemia)
		Inherited defect of intestinal iron absorption
		TMPRSS6 gene mutation (encoding matriptase-2)
		Disruption of potassium channel regulatory subunit KCNE2 in enterocytes
	Lack of vitamin B12	Diet
		Pernicious anemia (lack of intrinsic factor, gastric disease)
		Transcobalamin receptor KO
	Lack of folic acid	Diet
		Drug-induced lack of folic acid (5-fluorouracil)
		Virus-induced deficiency of folic acid
	Lack of EPO	EPO KO
		EPO deficiency
		Renal insufficiency
	Aplasia, bone marrow disorder	Radiation
		Myelodysplastic syndrome disease
		Viral infection (EBV, CMV)
		Drugs (phenylhydrazin)
Increased hemolysis or RBC depletion	Hemoglobinopathies	SCD
		Thalassemia (β-thalassemia intermedia and major)
		Hb-deficient mice
	Defects of cytoskeletal proteins	Spectrin gene mutations
		Ankyrin-deficient mice
		Erythroid cell-specific band 3 KO mice
	Defect of metabolic enzymes	Glucose-6-phosphate dehydrogenase deficiency
		Pyruvate kinase deficiency (glycolysis defect)
		Hexokinase 1 deficiency
	Disorder of redox regulation	SOD1 deficiency
		SOD2 deficiency
		Prx1 and Prx2 KO
		Nrf2 Trsp double KO
		Glutathione peroxidase 4 KO
	Antibody-related RBC membrane damage	Transfusion associated: blood groups allocated antibody production (ABO, Rh factor)
		Autoimmune: anti-RBC autoantibody transgenic mice
		Drug induced (L-dopa, phenylhydrazine)
		Injection of antimurine RBC antibodies (TER-119, 34-3C, 4C8)
		Warm antibody related (NZB/BL mice)
	Chronic inflammation	Infectious
		Tuberculosis (*Mycobacterium tuberculosis*)
		Brucellose (*Brucella abortus*)
		Malaria (*Plasmodium falciparum*)
		Trypanosomiasis (*Trypanosoma brucei*)
		Bacterial infections (*Staphylococcus aureus*, *Staphylococcus pyogenes*, *Helicobacter pylori etc*.)
		Pseudoinfectious
		Complete Freund's adjuvant injection (dried and inactivated mycobacteria, they cause an acute increase in hepcidin)
		Lipopolysaccharide injection (components of the outer cell membrane of gram-negative bacteria, which elicit a potent inflammatory response and increase hepcidin)
		Noninfectious
		Turpentine injection (induces an acute increase of hepcidin)
		Collagen injection (induces arthritis)
		Oral feeding of dextran sulfate sodium (induces colitis)
		Genetical
		IL-6-hepcidin-ferroportin axis
	Disorders of cytokine production	Transgenic expression of the IL-23 subunit p19
	Chronic disease/cancer	Acute and chronic colitis
		Systemic lupus erythematosus
		Lung cancer, melanoma, ovarian cancer
	Drugs (side effects)/poisoning	Anti-immune drugs
		Chemotherapeutical drugs (cisplatin, 5-fluorouracil, cyclophosphamid)
		Antiretroviral medication (zidovudine)
	Genetic factors	Paroxysmal nocturnal hemoglobinuria
		CD22 deficiency (defective allele of glucose phosphate isomerase, Gpi1c)
	Other nonspecific factors	CD47-deficient nonobese diabetic mice
		Gene disruption of dematin
		Extreme endurance exercise
		Genetical disorders (*e.g*., Fanconi anemia)
		kd/kd mice (knockdown mutation of Hif-2α)
		Ferrochelatase deficiency (mimics erythropoietic protoporphyria)
		Depurination of the 28S rRNA by ricin (induces hemolytic uremic syndrome)
Loss of RBCs	Blood loss	Repetitive phlebotomy
Disorder of RBC distribution	Hypersplenism	Adoptive transfer of syngeneic spleen cells

EPO, erythropoietin; KO, knockout; Nrf2, nuclear factor E2-related factor 2; RBC, red blood cell; SCD, sickle cell disease.

### Classification of anemic conditions

There are several ways to classify anemic conditions, of which the most common are based on the morphology/size of circulating RBCs (*i.e*., normocytic, macrocytic, and microcytic anemia), on the concentration of Hb in RBCs (*i.e*., normochromic, hypochromic, hyperchromic), or on etiopathology, that is, due to (I) defects in erythropoiesis; (II) increased hemolysis or RBC uptake by the reticuloendothelial system of the spleen; (III) bleeding; or (IV) a disorder of cell distribution (193) (Fig. 5). While an exhaustive description of all types of anemia is behind the scope of this review, examples of etiological classification of anemia are reported in Table 1, along with relevant well-characterized mouse models of the different aspects of the disease.

**FIG. 6.**
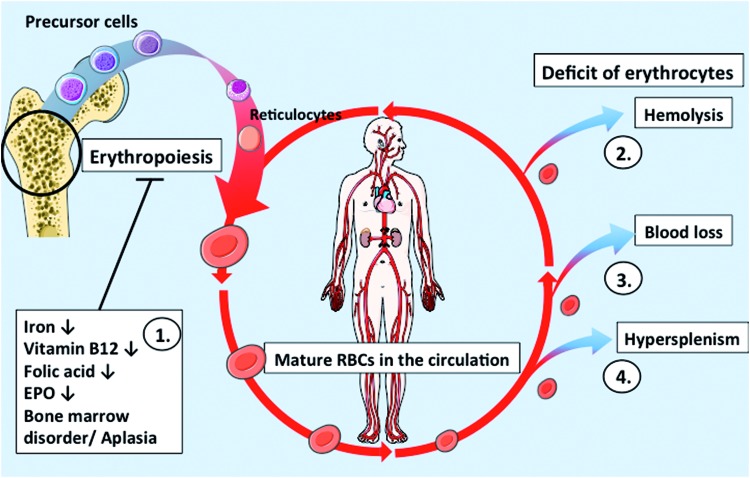
**RBC turnover and pathological changes in anemia. (1.)** Erythropoiesis occurs in the bone marrow, where blood cell precursors mature in ∼7 days. Iron, nutrient, or EPO deficiency, or other disorders of the bone marrow, negatively affect erythropoiesis leading to anemia. **(2.)** Hemolysis, *e.g.*, due to SCD, reduces the amount of functional circulating RBCs. **(3.)** Blood loss, due to repeated blood draws or a traumatic injury, can also lead to anemic conditions until new RBCs are formed. **(4.)** Hypersplenism can diminish the amount of circulating cells by increased RBC removal from the circulation. EPO, erythropoietin; SCD, sickle cell disease. To see this illustration in color, the reader is referred to the web version of this article at www.liebertpub.com/ars

Erythropoiesis can be impaired due to a lack of substrates needed for Hb synthesis (*e.g*., iron) or for cell division (*e.g*., vitamin B12 or folic acid), lack of erythropoietin (EPO) to stimulate erythropoiesis, or due to bone marrow disorders/aplasia. Beyond that decreases in circulating RBC numbers can be due to increased hemolysis or RBC depletion (Fig. 6).

Increased hemolysis may be caused by dysfunction of RBCs correlated with (I) defects of Hb (hemoglobinopathies) (18, 94, 161, 199), including alterations in structure and function of Hb chains; (II) defects of cytoskeletal proteins such as spectrin and ankyrin, which are essential for maintaining cell shape and integrity (19, 134, 149, 169); (III) defects in metabolic enzymes catalyzing the pentose phosphate cycle (126, 147) such as deficiency of G6PDH; (IV) redox dysregulation and defects of antioxidant enzymes (63, 84); (V) presence of antibodies directed against surface antigens on RBCs, such as antibodies anti-A, or anti-B blood group antigens (formed as a consequence of transfusion of allogenic blood from the wrong blood group), or autoimmune antibodies resulting in enhanced damage of RBC membranes (14, 92, 124, 160, 164, 178); (VI) chronic inflammation, disorders of cytokine production, and chronic diseases (97, 105, 154); (VII) side effects of drug/poisoning (32); (VIII) other genetic defects (155, 187); or (IX) RBC extrinsic factors (102, 108, 113, 142, 180). Another important cause of anemia is the loss of RBCs due to acute or chronic bleeding (59). Moreover, a disorder of cell distribution (for example, because of hypersplenism) may lead to anemia (Table 1). Please refer to Supplementary Data for references describing the mice models summarized in Supplementary Table S1 (Supplementary Data are available online at www.liebertpub.com/ars).

### RBC dysfunction and anemia

Dysfunction of intrinsic homeostatic mechanisms in RBCs may lead to anemic conditions both in humans and in mice. A number of different anemia conditions are due to RBC dysfunction induced by genetic defects as observed both in humans and in transgenic mice. These include (I) hemoglobinopathies, such as SCD, and thalassemia, which are well-described human genetic defects; (II) defects of cytoskeletal proteins, mainly described in transgenic mice; and (III) redox dysregulation related to genetic defects leading to increased production of oxidants and/or decreased antioxidant capacity, which ultimately lead to hemolytic anemia. Examples of genetic defects inducing redox dysregulation and hemolytic anemia are the genetic deficiency of G6PDH (well-characterized human disease) and the genetic deficiency of antioxidant enzymes, including SOD1 and 2, Prx1 and 2, and nuclear factor E2-related factor 2 (Nrf2, a transcription factor controlling expression of multiple antioxidant and detoxifying enzymes). Additionally, a lack of selenoproteins (*e.g*., GPx or Trx reductase) *via* removal of a tRNA [Sec] (Trsp) needed for selenocysteine utilization can cause decreased redox capacity (see also Table 1).

#### Hemoglobinopathies

##### Sickle cell disease

SCD is caused by a point mutation in the β Hb subunit chain and shows multiple clinical manifestations and early mortality, particularly if the individual is homozygous for the mutation or untreated (80). This mutation (Val6→Glu) causes the Hb tetramer to have different properties. The abnormal tetramer (called Hb S) is characterized by decreased solubility in deoxygenated conditions, and aggregates form into long chains. Accumulation of the long Hb S chains in an RBC causes a change in the shape (sickling) of RBCs, decreased deformability, and increased blood viscosity (34), resulting in peripheral vasoocclusion, a painful symptom. A sickle cell shape also negatively affects RBC deformability and prevents proper passage through capillary beds. It has been reported that sickle cells produce greater amounts of O_2_^•−^, H_2_O_2_, and hydroxyl radical ^•^OH compared with healthy RBCs (8, 80, 100). A major source of ROS in sickle RBCs is autoxidation of sickle cell Hb (80) and increase in NADPH oxidase activity (66). There are contradictive reports on decreased or increased activities of SOD1, Cat, and GPx (4, 41) in sickle RBCs. An increase in GSSG and decrease of GSH/GSSG ratios were reported in sickle RBCs, resulting in decreased antioxidative capacity. Increased consumption of antioxidants was also reported for other members of the nonenzymatic antioxidant system, including α-tocopherol and ascorbic acid (35, 86). Hydroxyurea is a cytostatic drug also applied in the therapy of SCD. Administration of hydroxyurea has been shown to induce expression of HbF, which interferes with the polymerization of HbS (33). A further consequence of sickling of cells is increased RBC hemolysis. Hemolysis affects not only the number of circulating RBCs but also release of Hb (and other cytoplasmic proteins) within the blood stream, thus profoundly affecting the viscosity and colloid properties of plasma and increasing systemic NO scavenging (71, 152).

##### Thalassemia

Thalassemia is another autosomal recessive group of genetic diseases resulting from defects in synthesis/structural properties of Hb subunits. The active oxygen-carrying form of Hb is a heterotetramer formed by two α and two β subunits. An excess of one subunit leads to denaturation of the excessive globin chain (165), followed by the formation of inclusion bodies (57). As in SCD, thalassemic patients show altered deformability as well as increased rigidity and instability of RBCs (166). Hb degradation leads to the release of heme and iron, which can serve as a catalyst for the Fenton reaction (165) (Fig. 2 and Eq. 3). This elevation of ROS levels is reflected in the decrease of GSH, increase of antioxidant enzymes, and occurrence of dysfunctional membrane proteins (31). Nagababu *et al.* (137) determined a 3.5-fold increase in heme degradation products in β-thalassemic mice and propose a less efficient antioxidant system within RBCs.

#### Defects of cytoskeletal proteins

RBC dysfunction leading to anemia may be also due to defects in cytoskeletal proteins (115, 127), as demonstrated by numerous transgenic mouse models lacking cytoskeletal proteins (see Fig. 3 for description of the cytoskeleton) (134). Homozygous mice carrying genes encoding for incomplete spectrin subunits result in severe anemia and dramatically decreased lifetime of RBCs (19). Ankyrin connects the spectrin cytoskeleton to the RBC membrane, specifically to band 3, a transmembrane anion exchanger channel. Deficiency of ankyrin in RBCs results in disruption of cytoskeletal formation, spherocytosis, and markedly increased mortality (149). Erythroid cell-specific knockout (KO) mice of band 3 induce changes in cell geometry, decreasing the cellular surface area-to-volume ratio, implying that the membrane/cytoskeleton interactions play a central role in defining RBC shape and functionality (169).

#### Redox dysregulation in RBCs and hemolytic anemia

There are few examples of genetic defects in humans leading to dysregulation of energetic and redox pathways leading to hemolytic anemia. One of the most common enzyme defects in humans is the deficiency of G6PDH. Additionally, there are several models of redox dysregulation induced by genetic deficiency of antioxidant enzymes, including SOD, Prx, and Nrf2/Trsp double-KO mouse models (see also Table 1), as described in detail below.

##### G6PDH deficiency

G6PDH deficiency is one of the most common enzyme defects in humans due to multiple mutations of the G6PDH gene and imparts different phenotypes, from acute hemolytic anemia to chronic hemolysis (30). Most G6PDH-deficient patients are asymptomatic throughout their lifetime and acute hemolytic anemia only occurs when triggered with certain drugs (antimalarial drugs such as primaquine), infections (hepatitis A virus), or pro-oxidative ingredients in food [*e.g*., fava beans, as already observed by the antiques (30)]. As described in the section “RBCs and Redox Regulation”, G6PDH is the enzyme that reduces NADP^+^ to NADPH as a branch of the glycolytic pathway. Deficiency of G6PDH induces a lack of reducing equivalents in RBCs and therefore decreases activity of GSH reductase, which reduces GSSG to GSH. A decrease in GSH and in GSH/GSSG ratio leads to a decrease in enzymatic activity of GPx and GRx, which use GSH as a cofactor (Fig. 2). Redox dysregulation in RBCs leads to membrane protein thiol oxidation and membrane damage, as well as oxidative damage of proteins, including Hb and cytoskeletal proteins in the spectrin–actin network (90). Oxidized, heme-depleted Hb forms precipitates (Heinz bodies), which accumulate within the cells, resulting in membrane rigidity and decreased RBC deformability (151).

##### SOD1 and SOD2 KO mice

Mice lacking the SOD1 gene show increased levels of ROS, decreased life span, increased reticulocyte counts, and increased levels of Heinz bodies, but overall these mice display a comparatively benign anemia and no changes in intracellular GSH levels (76). Although SOD2 is a mitochondrial enzyme and therefore existent in the RBC precursor cells only (118), SOD2 KO mice show severe RBC dysfunction and anemic phenotype characterized by decreased RBC deformability, increased heme degradation, and a reduced lifetime of RBCs (128).

##### Prx1 and Prx2 KO mice

Genetic deficiency of the cytosolic Prx1 in mice results in a severe anemic phenotype due to oxidative damage of the RBC membrane and cytosolic proteins (139). Mice with a deficiency of Prx2, the more abundant peroxiredoxin in RBCs, have decreased hematocrit levels and an increased amount of circulating reticulocytes (106). Since Prx2 is localized on/next to the membrane, these data suggest that with regard to SOD 1, scavenging of hydrogen peroxide in proximity to the membrane is more important than the interception of cytosolic hydrogen peroxide (106).

##### Nrf2/Trsp double-KO mice

The transcription factor Nrf2 is considered as a redox switch regulating the expression of antioxidant enzymes, GSH-synthesizing enzymes, and detoxifying Phase 2 enzymes (175), while the specific selenocysteine tRNA is needed for the synthesis of selenoproteins such as GPx or Trx reductase, which reduces oxidized Trx (133) (Fig. 2). Activation of Nrf2 was proposed to be a potential therapeutic target for the treatment of SCD and its activation decreased production of inflammatory cytokines and decreased the number of irregularly shaped erythrocytes (16). Nrf2 KO mice and Trsp KO mice are not anemic; but hemolytic anemia can be induced by treatment with oxidant drugs (54). In contrast, Nrf2/Trsp double-KO mice show a massive increase in ROS and severe anemia (95).

##### GPx4 KO mice

Isoform 4 of GPx (GPx4) is a mitochondrial enzyme important for detoxification of peroxides and H_2_O_2_ in mitochondria. GPx4 KO mice show impaired hematopoiesis without hemolysis (28), indicating that erythroid cells rather than adult RBCs are affected by deficient redox detoxification by GPx4.

### Changes in blood rheology and cardiovascular dysfunction in anemia

According to the classical view, anemia significantly affects tissue perfusion and oxygenation mainly *via* a decreased O_2_-carrying capacity of the blood (due to decreased Hb concentrations) and a change in the apparent viscosity of blood because of a decrease in the RBC number/concentration (78). Decrease in viscosity of blood reduces systemic vascular resistance and affects cardiovascular hemodynamics (20).

Investigations of the effects of acute normovolemic anemia induced by blood loss (*i.e*., without concomitant hemolysis) on cardiovascular hemodynamics were started almost 50 years ago by Fowler *et al.* (62), Murray and Rapaport (136), and Neill *et al.* (138). They showed that blood loss in healthy dogs increased heart rate, stroke volume, and cardiac output and decreased mean arterial pressure and systemic vascular resistance. Under chronic anemic conditions, adjustments that initially increase cardiac output lead to left ventricular enlargement and eccentric left ventricular hypertrophy (112). These changes are mainly due to increased preload, decreased afterload, and changes in cardiac geometry so that left ventricular systolic dysfunction may develop over time in patients without CVD. In addition, a pre-existing heart failure may be aggravated due to chronic anemia (7, 93). It was proposed that an increased sympathetic activity and an altered stimulation of the renin–angiotensin–aldosterone system may also contribute to the hyperdynamic state in anemia. Taken together, anemia leads to a persistent increase in cardiac workload to improve oxygen supply to organs and tissues. This persistent increase in cardiac work may induce progressive damage of cardiomyocytes, possibly leading to cardiac remodeling and to impairment of myocardial performance.

In addition to the well-known changes in cardiovascular hemodynamics in anemia, changes in intrinsic properties of RBCs may contribute to the systemic effects observed in anemia. Depending on the type of anemia, changes in viscosity may be correlated with changes in deformability (79) or changes in RBC-mediated NO metabolism. For example, in hemolytic anemia, release of free Hb may lead to systemic NO scavenging, affecting vascular tone (71, 152). On the contrary, in anemic conditions where the number of circulating RBCs is decreased without concomitant hemolysis, a decrease of RBC-dependent scavenging of NO may instead increase systemic NO bioavailability and cause vasodilation, increased flow, and may affect cardiovascular hemodynamics (140).

The role of NO metabolism in anemia in humans was studied by Anand *et al.* (7) in a seminal work; they found that patients with chronic severe anemia under basal conditions showed increased forearm blood flow compared with control patients. After treatment with the NOS inhibitor, N^G^-monomethyl-L-arginine, a threefold greater decrease of blood flow was detected in anemic patients compared with healthy controls and it was proposed that under anemic conditions, NO bioavailability may be increased by a decreased Hb-dependent inhibition of NO inactivation. They proposed that increase in NO bioavailability would increase cardiac output and thereby affect cardiovascular hemodynamics. In a rat model of iron deficiency anemia, expression and activity of eNOS were increased in the aorta and in the kidney (140), pointing to a further mechanism leading to increase in NO bioavailability in anemia. Effects of blood viscosity on shear stress-induced activation of eNOS in the endothelium were also proposed (119); therefore, a decrease in blood viscosity may also reduce endothelial-derived NO production and decrease NO bioavailability.

### Summary: RBC dysfunction and anemia

In conclusion, loss of cell integrity and RBC dysfunction in anemia are intimately interconnected with regulation of blood viscosity, peripheral resistance, cardiac and vascular function, also *via* control of NO metabolism and redox regulation. Therefore, we propose that intrinsic RBC dysfunction in anemia is not only a simple bystander/comorbidity of CVD but also significantly contributes to the appearance of severe complications and increased mortality as observed in the clinical setting.

## Anemia and CVD: Clinical Aspects and Therapeutic Approaches

Anemia is a frequent comorbidity in CVD (48) and is associated with higher mortality as well as increased hospitalization after myocardial infarction (91), even under mild anemic conditions (13, 156). There is accumulating evidence that anemia is related to a series of severe complications in CVD such as thromboembolic events (*e.g*., venous thrombosis and stroke), bleeding complications (191), uncontrolled hypertension (117), more frequent supraventricular and ventricular arrhythmias, and inflammation characterized by elevated levels of inflammatory cytokines (170). The underlying mechanisms of these complications are largely unidentified so far.

### Anemia in CVD and aging

There is compelling evidence that elderly patients—aged 65 years and older—have a 10% higher risk for the development of anemia, which increases to 20% in those aged 85 years and older (77). In general, the most frequent etiologies of anemia in elderly patients with CVD are nutritional deficiencies (*e.g*., of iron, vitamin B12, and folic acid) and chronic diseases, in particular renal insufficiency [leading to EPO deficiency (56)] and chronic inflammation. Moreover, a high percentage (almost 30%) of these patients exhibit idiopathic anemia (77) that may be caused by blood loss due to repetitive blood tests and surgical interventions, as well as occult bleeding (mainly in the gastrointestinal tract) during hospitalization. Moreover, the myelodysplastic syndrome (also defined as aplastic anemia) was proposed to be a frequent cause of idiopathic anemia (77); however, myelodysplastic syndrome is a rare disease, which mostly emerges initially in elderly patients who display alterations in blood cell counts (135). Taking in consideration the increasing aging of overall population, particularly in Western countries, understanding the role of anemia in CVD acquires increasing significance in clinical routine.

### Bleeding complications in anemia: RBC-PLT interactions

Increasing evidence exists that anemic patients and patients with coronary artery disease exhibit an elevated risk of major bleeding during hospitalization (48, 132, 174). It was proposed that functional alterations of PLTs (especially aggregability and PLT-endothelial interactions) in anemia (132) or the use of PLT aggregation-inhibiting medication (70) may affect blood flow and viscosity leading to an increased bleeding propensity.

Little is known about the interaction of RBCs and PLTs in the circulation and whether their interplay is disturbed in anemia, possibly resulting in enhanced bleeding complications as well as ischemic and thromboembolic events. NO is a potent inhibitor of PLT aggregation, acting *via* activation of soluble guanylyl cyclase in PLTs (64, 65). Therefore, one possibility to explain increased bleeding in anemic conditions without concomitant hemolysis is that a decreased number of RBCs may decrease NO scavenging and increase NO bioavailability, thereby inhibiting PLT aggregation and leading to bleeding complications (11, 65). A further mechanism proposed recently is that nitrite-derived NO release by RBCs under hypoxic conditions may contribute to hemostasis *via* inhibition of PLT aggregation (2, 144). On the contrary, in hemolytic anemia, increased NO scavenging by free Hb might decrease NO availability and therefore increase PLT aggregation and thromboembolism. Experimental studies demonstrated that PLTs can be activated *via* pharmacological inhibition of endogenous NO production in humans (159), leading to hypercoagulability and thrombotic complications (186). Furthermore, the risk of thromboembolism is elevated after transfusion of packed/damaged RBCs, which correlates with impaired outcome and survival of patients (47).

RBCs were also proposed to participate in thrombus formation by a direct physical interaction between PLTs and RBCs and/or damaged endothelial cells *via* interaction of membrane proteins/receptors (*e.g*., *via* integrins) or *via* exposition of PS on the surface of RBCs (23, 182). Valles *et al.* postulated that RBCs are able to modulate PLT aggregability and recruitment by enhancing α(IIb)β(3) integrin receptor activation and P-selectin expression on PLTs (182). In addition, PS on the outer leaflet of RBCs was identified to be a target mediator of PLT activation, resulting in enhanced aggregation (23). Notably, PS exposure on RBCs is increased in SCD (195).

Another possible coexisting mechanism involved in prothrombotic processes may be caused by increased oxidative stress in PLTs. There is compelling evidence that PLTs and RBCs from patients with different types of anemia such as SCD, β-thalassemia, and myelodysplastic syndrome show a dysregulation of redox systems (5, 6, 69) and altered PLT-RBC interactions. In particular, experimental studies indicate that elevated oxidative stress can be found in PLTs when incubated with thalassemic RBCs compared with normal RBCs, leading to increased PLT activation (5).

Taken together, clinical and experimental evidence reveals a complex role for RBCs in homeostasis and thrombosis, implicating RBC-PLT interactions, which involve membrane adhesion molecules, NO metabolism, and redox equilibria. Further research should address whether decreasing RBC number or a change in noncanonical functions of RBCs (such as control of NO metabolism and redox regulation) may account for alterations of RBC-PLT interactions and thrombotic and hemostatic complications in anemia.

### Therapeutic approaches: effects of blood transfusion in anemia

Blood transfusion is still the most important life-saving approach for critically ill patients with severe anemia, but a restrictive transfusion strategy should be followed according to current guidelines. However, there is compelling evidence that patients with CVD, treated acutely by blood transfusion, have a worsened outcome and show adverse events such as thromboembolism, allergic reactions, and fever, in addition to acute hemodynamic changes (such as elevated blood pressure and increased total blood volume and left ventricular work) (158). In case of repeated blood transfusions, additional transfusion-related complications arise, including acute lung injury, electrolyte disorders (hyperkalemia and hypocalcemia), and iron overload.

This adverse effects of transfusion might be due, in part, to a *storage lesion* of older RBC preparations, *i.e.*, changes of biochemical and molecular characteristics of RBCs during their storage, resulting in membrane damage and impaired function of RBCs. These changes include decreases in 2,3-diphosphoglycerate and ATP concentrations in stored RBCs, which result in decreased O_2_ delivery. In addition, storage of RBCs may affect also noncanonical function of RBCs, leading to decreased RBC deformability, impaired redox regulation, decrease of NO metabolites and increased membrane fragility, and thus to hemolysis and release of free Hb.

Free Hb seems to be one of the major pathophysiological issues of transfusion. An experimental study in guinea pigs demonstrated that treatment with the Hb scavenger haptoglobin ameliorates vascular dysfunction and heme-driven oxidative reactions (9). More recently, it was shown that stored RBCs release membrane fragments, or microparticles, containing Hb, which are able to scavenge NO and decrease endothelial function (153).

These findings imply that transfusion of older blood may profoundly affect cardiovascular hemodynamics and increase mortality by decreasing NO bioavailability. Therefore, understanding noncanonical functions of RBCs, and in particular the role of RBCs in NO metabolism, may provide a basis for development of new approaches to increase the safety and effectiveness of blood transfusion.

### Therapeutic approaches: effects of ESA administration in anemia

The administration of EPO or ESAs is a common therapeutic approach applied to stimulate and support erythropoiesis, particularly in patients with anemia associated with end-stage renal disease (21), patients with nonmyeloid cancer on chemotherapy, patients with HIV treated with zidovudine (58), or patients undergoing an elective, noncardiac nonvascular surgery to reduce the need for allogeneic blood transfusions (55). However, this therapeutic approach has its limitations. An estimated 10% of patients have inadequate responses to EPO or ESAs. EPO therapy can only be effective if the EPO responsivness in the bone marrow is still intact, EPO is not removed from the circulation (*via* receptor-mediated endocytosis, followed by degradation in lysosomes) (74), or if EPO can reach its receptor on the bone marrow and if the receptors and signaling pathways downstream can be activated. Moreover, a negative correlation between Hb levels and inflammatory markers was found in poor EPO responders, indicating that the severity of anemia correlates with the state of inflammation (176).

It is important to consider that the receptors for EPO are found not only in erythroid cells but also in other cell types, including cardiomyocytes, vascular cells, and mature RBCs. In fact, it was shown that EPO has off-target effects, particularly in the heart and in the vessels. Indeed, it was shown that EPO administration to normovolemic anemic patients increased systemic vascular resistance and arterial blood pressure and decreased cardiac output due to diminished heart rate and venous return (103, 194). The acute hemodynamic responses of EPO administration may result in impaired perfusion/oxygen supply to the tissues and contribute to acute adverse events such as enhanced PLT aggregation, thromboembolism, and myocardial ischemia (17, 45). Randomized clinical studies in patients with CVD demonstrate that EPO administration increases the mortality after cardiovascular events (143). However, experimental studies showed that administration of EPO under nonanemic conditions protects cardiomyocytes against ischemic injury *in vitro*, limits infarct size, and improves myocardial function after I/R injury (145).

### Conclusion: therapeutic challenges

From a clinical point of view, the increasing age of the overall population is accompanied by new challenges. In particular, one challenge is that 20%–30% of patients with CVD have concomitant anemia and that anemia itself may contribute to clinical complications and death. The therapeutic approaches currently applied in clinical practice not appear to be beneficial for these patients and, in some cases such as in transfusion of stored blood, may increase risk of mortality. Altered NO metabolism and NO scavenging by transfusion of damaged RBCs was proposed to play a role in cardiovascular effects of older blood, but alteration of other noncanonical functions of RBCs, including redox dysregulation and mechanical properties, may be involved in these effects.

A deeper understanding of the cause/effects of these cellular processes may allow establishing new prognostic and therapeutic procedures aimed to treat these conditions.

## Summary and Open Questions

There is accumulating evidence that RBCs, besides their function as O_2_/CO_2_ carriers and a pH control system, exert multiple noncanonical functions. These include a complex role in NO metabolism (*via* scavenging, transporting, and releasing NO and its metabolites), a systemic redox buffer contributing to maintain systemic redox regulation, and transport and release vasoactive molecules (*e.g*., ATP and NO/NO metabolites, defined as erythrocrine function). Additionally, according to basic science studies, RBCs may control systemic hemodynamics by influencing blood rheological properties and contribute to tissue protection and cardiovascular homeostasis (40, 73, 82).

Anemia is a pathological condition characterized by a decreased number of circulating RBCs. Despite its simple definition, anemia is characterized by multiple etiologies, phenotype of circulating RBCs, and pathophysiological outcome. Interestingly, there are big cohort/epidemiological studies showing associations between anemia and cardiovascular complications (48, 87, 91). Taking into account the overwhelming body of evidence of novel functional role of RBCs in the cardiovascular system, it is tempting to put forward the hypothesis that anemia may not be a simple bystander in CVD, but may contribute to worsening the outcome. Before establishing this, there are a number of open questions, which need to be answered first.

For example, it is not known whether the complications related to anemia are simply due to a decrease in the number/concentration of circulating RBCs (inducing a systemic dysfunction of normal RBCs) or are rather due to an intrinsic RBC dysfunction (*e.g*., less efficient redox buffering, decreased NO production, increased RBC fragility) or both. To test this important distinction experimentally, functional properties of RBCs from anemic patients should be compared with normal aged-matched patients with and without concomitant CVD, and blood from clinical relevant anemic models should be transferred to nonanemic models, and vice versa. These (and similar) experiments, together with interventional clinical studies, may reveal whether anemia should be to be considered as an epiphenomenon, a bystander, a risk indicator, or even a risk factor of mortality in CVD.

Moreover, to understand the role of canonical and noncanonical functions of RBCs in anemia associated with CVD, it will be necessary to carefully distinguish between the different forms of anemia and their consequences on RBC structural and functional characteristics. For example, while effects of blood loss may be strictly linked to effects of decreased RBC count and volume, the pathological consequences of hemolytic anemia (which is mainly linked to a dysregulation of redox homeostasis or to hemoglobinopathies) may be related more closely to massive release of Hb in the circulation and its NO-scavenging properties. This is particularly important for choosing an appropriate experimental setting and/or animal model, which should reflect the characteristics of the anemic condition under study (*e.g*., anemia due to blood loss, inefficient erythropoiesis, hemoglobinopathy, or hemolysis). An overview of the current models can be found in Table 1.

However, it is important to point out that integration of basic or animal experiments with epidemiological studies is difficult and should be done with extreme caution. Interpretation of the results from big cohort/epidemiological studies requires recognition of many features of the disease and normal physiology, which are interacting. Nevertheless, analysis of epidemiological observations allows identifying possible interrelations between the observed phenomena as well as new open questions, helping to formulate specific working/testable hypotheses and define new research directions.

We believe that evaluation of pathophysiological changes in RBC functions in anemia associated with cardiovascular and other related disease conditions both in relevant animal models and clinical settings will be critical to understand whether the observed increase in morbidity and mortality in patients CVD with concomitant anemia has a mechanistic foundation and specifically may be related to an intrinsic or systemic impairment of noncanonical functions of RBCs. This may provide novel therapeutic strategies to decrease morbidity and mortality in CVD with concomitant anemia.

## Supplementary Material

Supplemental data

## Abbreviations Used

ATP: adenosine triphosphate
cAMP: cyclic adenosine monophosphate
Cat: catalase
CO_2_: carbon dioxide
CVD: cardiovascular disease
ECs: endothelial cells
eNOS: endothelial nitric oxide synthase
EPO: erythropoietin
ESA: erythropoiesis-stimulating agent
FAD: flavin adenine dinucleotide
G6PDH: glucose-6-phosphate dehydrogenase
GPx: glutathione peroxidase
Gpx4: glutathione peroxidase 4
GRx: glutaredoxin
GSH: glutathione
GSSG: glutathione disulfide
Hb: hemoglobin
KO: knockout
MCH: mean corpuscular hemoglobin
metHb: methemoglobin
NADH: nicotinamide adenine dinucleotide
NADPH: nicotinamide adenine dinucleotide phosphate
NO: nitric oxide
NOS: nitric oxide synthase
Nrf2: nuclear factor E2-related factor 2
PLT: platelet
PMRS: plasma membrane redox system
Prx2: peroxiredoxin 2
PS: phosphatidylserine
RBC: red blood cell
ROS: reactive oxygen species
SCD: sickle cell disease
SOD1: superoxide dismutase 1
Trsp: tRNA[Sec]
Trx: thioredoxin
VSMC: vascular smooth muscle cell
