# Supplementary material for: Red Blood Cell Function and Dysfunction: Redox Regulation, Nitric Oxide Metabolism, Anemia
Source: Antioxid Redox Signal. 2017 May 1;26(13):718–42. doi: 10.1089/ars.2016.6954 (PMC5421513; doi:10.1089/ars.2016.6954)
Supplement: Supplemental data [file Supp_Table1.pdf]

Supplementary Data

Supplementary Table S1. Etiological Classification of Anemia and Characterized Mice Models, Including References

Disorder of erythropoiesis	Iron deficiency	Diet (36)<br>Genetical: Trf(hpx/hpx) gene mutation (hypotransferrinemia) (55)<br>Inherited defect of intestinal iron absorption (1)<br>TMPRSS6 gene mutation (encoding matriptase-2) (11)<br>Disruption of potassium channel regulatory subunit KCNE2 in enterocytes (47)
	Lack of vitamin B12	Diet (15)<br>Pernicious anemia (lack of intrinsic factor, gastric disease) (64)<br>Transcobalamin receptor KO (27)
	Lack of folic acid	Diet (6)<br>Drug-induced lack of folic acid (5-fluorouracil) (20)<br>Virus-induced deficiency of folic acid (26)
	Lack of erythropoietin	EPO KO (60)<br>EPO deficiency (61)<br>Renal insufficiency (39)
	Aplasia, bone marrow disorder	Radiation (48)<br>Myelodysplastic syndrome disease (63)<br>Viral infection (EBV, CMV) (48)<br>Drugs (phenylhydrazin) (53)
Increased hemolysis or RBC depletion	Hemoglobinopathies	SCD (5)<br>Thalassemia (β-thalassemia intermedia and major) (21, 50)<br>Hb-deficient mice (62)
	Defects of cytoskeletal proteins	Spectrin gene mutations (7)<br>Ankyrin-deficient mice (42)<br>Erythroid cell-specific band 3 KO mice (52)
	Defect of metabolic enzymes	Glucose-6-phosphate dehydrogenase deficiency (44)<br>Pyruvate kinase deficiency (glycolysis defect) (35)<br>Hexokinase 1 deficiency (41)
	Disorder of redox regulation	SOD1 deficiency (17)<br>SOD2 deficiency (13)<br>Prx1 and Prx2 KO (30, 38)<br>Nrf2 Trsp double KO (22)<br>Glutathione peroxidase 4 KO (50)
	Antibody-related RBC membrane damage	Transfusion-associated<br>Blood groups allocated antibody production (ABO, Rh factor) (16, 49, 54)<br>Autoimmune: anti-RBC autoantibody transgenic mice (3)<br>Drug-induced [L-dopa (51), phenylhydrazine (18)]<br>Injection of antimurine RBC antibodies (TER-119, 34-3C, 4C8) (19)<br>Warm antibody related (NZB/BL mice) (34)
	Chronic inflammation	Infectious<br>Tuberculosis (*Mycobacterium tuberculosis*) (43)<br>Brucellose (*Brucella abortus*) (23)<br>Malaria (*Plasmodium falciparum*) (28)<br>Trypanosomiasis (*Trypanosoma brucei*) (43)<br>Bacterial infections (*Staphylococcus aureus*, *Staphylococcus pyogenes*, *H. pylori*, *etc.*) (8, 58)<br>Pseudoinfectious<br>Complete Freund's adjuvant injection (dried and inactivated mycobacteria, they cause an acute increase in hepcidin) (43)<br>Lipopolysaccharide injection (components of the outer cell membrane of gram-negative bacteria, which elicit a potent inflammatory response and increase hepcidin) (29)<br>Noninfectious<br>Turpentine injection (induces an acute increase of hepcidin) (43)<br>Collagen injection (induces arthritis) (43)<br>Oral feeding of dextran sulfate sodium (induces colitis) (43)<br>Genetical<br>IL-6-hepcidin-ferroportin axis (37)

(*continued*)

	Disorders of cytokine production	Transgenic expression of the IL-23 subunit p19 (4)
	Chronic diseases/ cancer	Acute and chronic colitis (9)<br>Systemic lupus erythematosus (14)<br>Lung cancer, melanoma, ovarian cancer (24)
	Drug side effects/ poisoning	Anti-immune drugs (48)<br>Chemotherapeutical drugs (cisplatin, 5-fluorouracil, cyclophosphamide) (2, 20)<br>Antiretroviral medication (zidovudine) (10)
	Genetic factors	Paroxysmal nocturnal hemoglobinuria (45)<br>CD22 deficiency (defective allele of glucose phosphate isomerase, Gpi1c) (59)
	Other nonspecific factors	CD47-deficient nonobese diabetic mice (40)<br>Gene disruption of dematin (32)<br>Extreme endurance exercise (31)<br>Genetical disorders (e.g., Fanconi anemia)<br>kd/kd mice (knockdown mutation of Hif-2α) (56)<br>Ferrochelatase deficiency (mimics erythropoietic protoporphyria) (33)<br>Depurination of the 28S rRNA by ricin (induces hemolytic uremic syndrome) (25)
Loss of RBCs	Blood loss	Repetitive phlebotomy (12, 57)
Disorder of RBC distribution	Hypersplenism	Adoptive transfer of syngeneic spleen cells (46)

EPO, erythropoietin; KO, knockout; Nrf2, nuclear factor E2-related factor 2; RBC, red blood cell; SCD, sickle cell disease.

Supplementary References

1. Anderson GJ, Murphy TL, Cowley L, Evans BA, Halliday JW, and McLaren GD. Mapping the gene for sex-linked anemia: an inherited defect of intestinal iron absorption in the mouse. *Genomics* 48: 34–39, 1998.
2. Bartucci M, Dattilo R, Martinetti D, Todaro M, Zapparelli G, Di Virgilio A, Biffoni M, De Maria R, and Zeuner A. Prevention of chemotherapy-induced anemia and thrombocytopenia by constant administration of stem cell factor. *Clin Cancer Res* 17: 6185–6191, 2011.
3. Baudino L, Fossati-Jimack L, Chevalley C, Martinez-Soria E, Shulman MJ, and Izui S. IgM and IgA anti-erythrocyte autoantibodies induce anemia in a mouse model through multivalency-dependent hemagglutination but not through complement activation. *Blood* 109: 5355–5362, 2007.
4. Beurlet S, Chomienne C, and Padua RA. Engineering mouse models with myelodysplastic syndrome human candidate genes; how relevant are they? *Haematologica* 98: 10–22, 2013.
5. Beuzard Y. Mouse models of sickle cell disease. *Transfus Clin Biol* 15: 7–11, 2008.
6. Bills ND, Koury MJ, Clifford AJ, and Dessypris EN. Ineffective hematopoiesis in folate-deficient mice. *Blood* 79: 2273–2280, 1992.
7. Birkenmeier CS, McFarland-Starr EC, and Barker JE. Chromosomal location of three spectrin genes: relationship to the inherited hemolytic anemias of mouse and man. *Proc Natl Acad Sci U S A* 85: 8121–8125, 1988.
8. Burns M, Muthupalani S, Ge Z, Wang TC, Bakthavatchalu V, Cunningham C, Ennis K, Georgieff M, and Fox JG. *Helicobacter pylori* infection induces anemia, depletes serum iron storage, and alters local iron-related and adult brain gene expression in male INS-GAS mice. *PLoS One* 10: e0142630, 2015.
9. Carter PR, Watts MN, Kosloski-Davidson M, Prasai K, Grisham MB, and Harris NR. Iron status, anemia, and plasma erythropoietin levels in acute and chronic mouse models of colitis. *Inflamm Bowel Dis* 19: 1260–1265, 2013.
10. Chow FP and Hamburger AW. In vivo evaluation of the anemia induced by azidothymidine (AZT) in a murine model of AIDS. *Eur J Haematol* 47: 91–97, 1991.
11. De Falco L, Sanchez M, Silvestri L, Kannengiesser C, Muckenthaler MU, Iolascon A, Gouya L, Camaschella C, and Beaumont C. Iron refractory iron deficiency anemia. *Haematologica* 98: 845–853, 2013.
12. Foller M, Feil S, Ghoreschi K, Koka S, Gerling A, Thunemann M, Hofmann F, Schuler B, Vogel J, Pichler B, Kasinathan RS, Nicolay JP, Huber SM, Lang F, and Feil R. Anemia and splenomegaly in cGKI-deficient mice. *Proc Natl Acad Sci U S A* 105: 6771–6776, 2008.
13. Friedman JS, Rebel VI, Derby R, Bell K, Huang TT, Kuypers FA, Epstein CJ, and Burakoff SJ. Absence of mitochondrial superoxide dismutase results in a murine hemolytic anemia responsive to therapy with a catalytic antioxidant. *J Exp Med* 193: 925–934, 2001.
14. Fujii J, Kurahashi T, Konno T, Homma T, and Iuchi Y. Oxidative stress as a potential causal factor for autoimmune hemolytic anemia and systemic lupus erythematosus. *World J Nephrol* 4: 213–222, 2015.
15. Ghosh S, Sinha JK, Putcha UK, and Raghunath M. Severe but not moderate vitamin B12 deficiency impairs lipid profile, induces adiposity, and leads to adverse gestational outcome in female C57BL/6 mice. *Front Nutr* 3: 1, 2016.
16. Hod EA, Zimring JC, and Spitalnik SL. Lessons learned from mouse models of hemolytic transfusion reactions. *Curr Opin Hematol* 15: 601–605, 2008.
17. Iuchi Y, Okada F, Takamiya R, Kibe N, Tsunoda S, Nakajima O, Toyoda K, Nagae R, Suematsu M, Soga T, Uchida K, and Fujii J. Rescue of anaemia and autoimmune

responses in SOD1-deficient mice by transgenic expression of human SOD1 in erythrocytes. *Biochem J* 422: 313–320, 2009.

18. Jiang X, Gao M, Chen Y, Liu J, Qi S, Ma J, Zhang Z, and Xu Y. EPO-dependent induction of erythroferrone drives hepcidin suppression and systematic iron absorption under phenylhydrazine-induced hemolytic anemia. *Blood Cells Mol Dis* 58: 45–51, 2016.
19. Jordan MB, van Rooijen N, Izui S, Kappler J, and Marrack P. Liposomal clodronate as a novel agent for treating autoimmune hemolytic anemia in a mouse model. *Blood* 101: 594–601, 2003.
20. Kalechman Y, Rushkin G, Nerubay J, Albeck M, and Sredni B. Effect of the immunomodulator AS101 on chemotherapy-induced multilineage myelosuppression, thrombocytopenia, and anemia in mice. *Exp Hematol* 23: 1358–1366, 1995.
21. Kautz L, Jung G, Du X, Gabayan V, Chapman J, Nasoff M, Nemeth E, and Ganz T. Erythroferrone contributes to hepcidin suppression and iron overload in a mouse model of beta-thalassemia. *Blood* 126: 2031–2037, 2015.
22. Kawatani Y, Suzuki T, Shimizu R, Kelly VP, and Yamamoto M. Nrf2 and selenoproteins are essential for maintaining oxidative homeostasis in erythrocytes and protecting against hemolytic anemia. *Blood* 117: 986–996, 2011.
23. Kim A, Fung E, Parikh SG, Valore EV, Gabayan V, Nemeth E, and Ganz T. A mouse model of anemia of inflammation: complex pathogenesis with partial dependence on hepcidin. *Blood* 123: 1129–1136, 2014.
24. Kim A, Rivera S, Shprung D, Limbrick D, Gabayan V, Nemeth E, and Ganz T. Mouse models of anemia of cancer. *PLoS One* 9: e93283, 2014.
25. Korcheva V, Wong J, Corless C, Iordanov M, and Magun B. Administration of ricin induces a severe inflammatory response via nonredundant stimulation of ERK, JNK, and P38 MAPK and provides a mouse model of hemolytic uremic syndrome. *Am J Pathol* 166: 323–339, 2005.
26. Koury MJ and Horne DW. Apoptosis mediates and thymidine prevents erythroblast destruction in folate deficiency anemia. *Proc Natl Acad Sci U S A* 91: 4067–4071, 1994.
27. Lai SC, Nakayama Y, Sequeira JM, Wlodarczyk BJ, Cabrera RM, Finnell RH, Bottiglieri T, and Quadros EV. The transcobalamin receptor knockout mouse: a model for vitamin B12 deficiency in the central nervous system. *FASEB J* 27: 2468–2475, 2013.
28. Lamikanra AA, Brown D, Potocnik A, Casals-Pascual C, Langhorne J, and Roberts DJ. Malarial anemia: of mice and men. *Blood* 110: 18–28, 2007.
29. Lasocki S, Millot S, Andrieu V, Letteron P, Pilard N, Muzeau F, Thibaudeau O, Montravers P, and Beaumont C. Phlebotomies or erythropoietin injections allow mobilization of iron stores in a mouse model mimicking intensive care anemia. *Crit Care Med* 36: 2388–2394, 2008.
30. Lee TH, Kim SU, Yu SL, Kim SH, Park DS, Moon HB, Dho SH, Kwon KS, Kwon HJ, Han YH, Jeong S, Kang SW, Shin HS, Lee KK, Rhee SG, and Yu DY. Peroxiredoxin II is essential for sustaining life span of erythrocytes in mice. *Blood* 101: 5033–5038, 2003.
31. Liu QS, Wang JH, Cui J, Yang ZH, and Du GH. A novel acute anemia model for pharmacological research in mice by compelled acute exercise. *Acta Pharmacol Sin* 30: 1643–1647, 2009.
32. Lu Y, Hanada T, Fujiwara Y, Nwankwo JO, Wieschhaus AJ, Hartwig J, Huang S, Han J, and Chishti AH. Gene disruption of dematin causes precipitous loss of erythrocyte membrane stability and severe hemolytic anemia. *Blood* 128: 93–103, 2016.
33. Lyoumi S, Abitbol M, Andrieu V, Henin D, Robert E, Schmitt C, Gouya L, de Verneuil H, Deybach JC, Montagutelli X, Beaumont C, and Puy H. Increased plasma transferrin, altered body iron distribution, and microcytic hypochromic anemia in ferrochelatase-deficient mice. *Blood* 109: 811–818, 2007.
34. Mellors RC. Autoimmune disease in NZB/BL Mice. I. Pathology and pathogenesis of a model system of spontaneous glomerulonephritis. *J Exp Med* 122: 25–40, 1965.
35. Min-Oo G, Fortin A, Tam MF, Nantel A, Stevenson MM, and Gros P. Pyruvate kinase deficiency in mice protects against malaria. *Nat Genet* 35: 357–362, 2003.
36. Nagababu E, Gulyani S, Earley CJ, Cutler RG, Mattson MP, and Rifkind JM. Iron-deficiency anaemia enhances red blood cell oxidative stress. *Free Radic Res* 42: 824–829, 2008.
37. Nemeth E, Valore EV, Territo M, Schiller G, Lichtenstein A, and Ganz T. Hepcidin, a putative mediator of anemia of inflammation, is a type II acute-phase protein. *Blood* 101: 2461–2463, 2003.
38. Neumann CA, Krause DS, Carman CV, Das S, Dubey DP, Abraham JL, Bronson RT, Fujiwara Y, Orkin SH, and Van Etten RA. Essential role for the peroxiredoxin Prdx1 in erythrocyte antioxidant defence and tumour suppression. *Nature* 424: 561–565, 2003.
39. Ogino A, Takemura G, Kawasaki M, Tsujimoto A, Kanamori H, Li L, Goto K, Maruyama R, Kawamura I, Takeyama T, Kawaguchi T, Watanabe T, Moriguchi Y, Saito H, Fujiwara T, Fujiwara H, and Minatoguchi S. Erythropoietin receptor signaling mitigates renal dysfunction-associated heart failure by mechanisms unrelated to relief of anemia. *J Am Coll Cardiol* 56: 1949–1958, 2010.
40. Oldenborg PA, Gresham HD, Chen Y, Izui S, and Lindberg FP. Lethal autoimmune hemolytic anemia in CD47-deficient nonobese diabetic (NOD) mice. *Blood* 99: 3500–3504, 2002.
41. Peters LL, Lane PW, Andersen SG, Gwynn B, Barker JE, and Beutler E. Downeast anemia (dea), a new mouse model of severe nonspherocytic hemolytic anemia caused by hexokinase (HK(1)) deficiency. *Blood Cells Mol Dis* 27: 850–860, 2001.
42. Rank G, Sutton R, Marshall V, Lundie RJ, Caddy J, Romeo T, Fernandez K, McCormack MP, Cooke BM, Foote SJ, Crabb BS, Curtis DJ, Hilton DJ, Kile BT, and Jane SM. Novel roles for erythroid Ankyrin-1 revealed through an ENU-induced null mouse mutant. *Blood* 113: 3352–3362, 2009.
43. Rivera S and Ganz T. Animal models of anemia of inflammation. *Semin Hematol* 46: 351–357, 2009.
44. Rochford R, Ohrt C, Baresel PC, Campo B, Sampath A, Magill AJ, Tekwani BL, and Walker LA. Humanized mouse model of glucose 6-phosphate dehydrogenase deficiency for in vivo assessment of hemolytic toxicity. *Proc Natl Acad Sci U S A* 110: 17486–17491, 2013.
45. Rosti V. Murine models of paroxysmal nocturnal hemoglobinuria. *Ann N Y Acad Sci* 963: 290–296, 2002.
46. Salman H, Bessler H, Bergman M, Fibach E, and Djaldetti M. Functional hypersplenism in mice induced by adoptive transfer of syngeneic spleen cells. *Exp Biol Med (Maywood)* 231: 112–116, 2006.

47. Salsbury G, Cambridge EL, McIntyre Z, Arends MJ, Karp NA, Isherwood C, Shannon C, Hooks Y, Ramirez-Solis R, Adams DJ, White JK, and Speak AO. Disruption of the potassium channel regulatory subunit KCNE2 causes iron-deficient anemia. *Exp Hematol* 42: 1053–1058.e1, 2014.
48. Scheinberg P and Chen J. Aplastic anemia: what have we learned from animal models and from the clinic. *Semin Hematol* 50: 156–164, 2013.
49. Schirmer DA, Song SC, Baliff JP, Harbers SO, Clynes RA, Krop-Watorek A, Halverson GR, Czerwinski M, and Spitalnik SL. Mouse models of IgG- and IgM-mediated hemolysis. *Blood* 109: 3099–3107, 2007.
50. Schmidt PJ, Toudjarska I, Sendamarai AK, Racie T, Milstein S, Bettencourt BR, Hettinger J, Bumcrot D, and Fleming MD. An RNAi therapeutic targeting Tmprss6 decreases iron overload in Hfe(-/-) mice and ameliorates anemia and iron overload in murine beta-thalassemia intermedia. *Blood* 121: 1200–1208, 2013.
51. Sharon R and Naor D. Experimental model of autoimmune hemolytic anemia induced in mice with levodopa. *Clin Immunol Immunopathol* 52: 160–172, 1989.
52. Southgate CD, Chishti AH, Mitchell B, Yi SJ, and Palek J. Targeted disruption of the murine erythroid band 3 gene results in spherocytosis and severe haemolytic anaemia despite a normal membrane skeleton. *Nat Genet* 14: 227–230, 1996.
53. Spivak JL, Toretti D, and Dickerman HW. Effect of phenylhydrazine-induced hemolytic anemia on nuclear RNA polymerase activity of the mouse spleen. *Blood* 42: 257–266, 1973.
54. Tomita H, Fuchimoto Y, Mori T, Kato J, Uemura T, Handa M, Tazawa H, Ohdan H, Okamoto S, and Kuroda T. Production of anti-ABO blood group antibodies after minor ABO-incompatible bone marrow transplantation in NOD/SCID/gamma(c)(null) mice. *Clin Transplant* 27: E702–E708, 2013.
55. Trenor CC, 3rd, Campagna DR, Sellers VM, Andrews NC, and Fleming MD. The molecular defect in hypotransferrinemic mice. *Blood* 96: 1113–1118, 2000.
56. Tsuboi I, Yamashita T, Nagano M, Kimura K, To'a Salazar G, and Ohneda O. Impaired expression of HIF-2alpha induces compensatory expression of HIF-1alpha for the recovery from anemia. *J Cell Physiol* 230: 1534–1548, 2015.
57. Tsui AK, Marsden PA, Mazer CD, Adamson SL, Henkelman RM, Ho JJ, Wilson DF, Heximer SP, Connelly KA, Bolz SS, Lidington D, El-Beheiry MH, Dattani ND, Chen KM, and Hare GM. Priming of hypoxia-inducible factor by neuronal nitric oxide synthase is essential for adaptive responses to severe anemia. *Proc Natl Acad Sci U S A* 108: 17544–17549, 2011.
58. Valdes-Ferrer SI, Papoin J, Dancho ME, Olofsson P, Li J, Lipton JM, Avancena P, Yang H, Zou YR, Chavan SS, Volpe BT, Gardenghi S, Rivella S, Diamond B, Andersson U, Steinberg BM, Blanc L, and Tracey KJ. HMGB1 mediates anemia of inflammation in murine sepsis survivors. *Mol Med* 2015 [Epub ahead of print]; DOI: 10.2119/molmed.2015.00243.
59. Walker JA, Hall AM, Kotsopoulou E, Espeli M, Nitschke L, Barker RN, Lyons PA, and Smith KG. Increased red cell turnover in a line of CD22-deficient mice is caused by Gpi1c: a model for hereditary haemolytic anaemia. *Eur J Immunol* 42: 3212–3222, 2012.
60. Wu H, Lee SH, Gao J, Liu X, and Iruela-Arispe ML. Inactivation of erythropoietin leads to defects in cardiac morphogenesis. *Development* 126: 3597–3605, 1999.
61. Yamazaki S, Souma T, Hirano I, Pan X, Minegishi N, Suzuki N, and Yamamoto M. A mouse model of adult-onset anaemia due to erythropoietin deficiency. *Nat Commun* 4: 1950, 2013.
62. Zhang AS, Sheftel AD, and Ponka P. The anemia of ''haemoglobin-deficit'' (hbd/hbd) mice is caused by a defect in transferrin cycling. *Exp Hematol* 34: 593–598, 2006.
63. Zhou T, Kinney MC, Scott LM, Zinkel SS, and Rebel VI. Revisiting the case for genetically engineered mouse models in human myelodysplastic syndrome research. *Blood* 126: 1057–1068, 2015.
64. Zhou Z, Miao R, Huang S, Elder B, Quinn T, Papasian CJ, Zhang J, Fan D, Chen YE, and Fu M. MCPIP1 deficiency in mice results in severe anemia related to autoimmune mechanisms. *PLoS One* 8: e82542, 2013.
